# Repeated nebulisation of non-viral *CFTR* gene therapy in patients with cystic fibrosis: a randomised, double-blind, placebo-controlled, phase 2b trial

**DOI:** 10.1016/S2213-2600(15)00245-3

**Published:** 2015-09

**Authors:** Eric W F W Alton, David K Armstrong, Deborah Ashby, Katie J Bayfield, Diana Bilton, Emily V Bloomfield, A Christopher Boyd, June Brand, Ruaridh Buchan, Roberto Calcedo, Paula Carvelli, Mario Chan, Seng H Cheng, D David S Collie, Steve Cunningham, Heather E Davidson, Gwyneth Davies, Jane C Davies, Lee A Davies, Maria H Dewar, Ann Doherty, Jackie Donovan, Natalie S Dwyer, Hala I Elgmati, Rosanna F Featherstone, Jemyr Gavino, Sabrina Gea-Sorli, Duncan M Geddes, James S R Gibson, Deborah R Gill, Andrew P Greening, Uta Griesenbach, David M Hansell, Katharine Harman, Tracy E Higgins, Samantha L Hodges, Stephen C Hyde, Laura Hyndman, J Alastair Innes, Joseph Jacob, Nancy Jones, Brian F Keogh, Maria P Limberis, Paul Lloyd-Evans, Alan W Maclean, Michelle C Manvell, Dominique McCormick, Michael McGovern, Gerry McLachlan, Cuixiang Meng, M Angeles Montero, Hazel Milligan, Laura J Moyce, Gordon D Murray, Andrew G Nicholson, Tina Osadolor, Javier Parra-Leiton, David J Porteous, Ian A Pringle, Emma K Punch, Kamila M Pytel, Alexandra L Quittner, Gina Rivellini, Clare J Saunders, Ronald K Scheule, Sarah Sheard, Nicholas J Simmonds, Keith Smith, Stephen N Smith, Najwa Soussi, Samia Soussi, Emma J Spearing, Barbara J Stevenson, Stephanie G Sumner-Jones, Minna Turkkila, Rosa P Ureta, Michael D Waller, Marguerite Y Wasowicz, James M Wilson, Paul Wolstenholme-Hogg, UK Cystic Fibrosis Gene Therapy Consortium

**Affiliations:** a Royal Brompton and Harefield NHS Foundation Trust, London, UK; b Imperial College London, London, UK; c Western General Hospital, Edinburgh, UK; d Usher Institute of Population Health Sciences and Informatics and Centre for Population Health Sciences, University of Edinburgh, Edinburgh, UK; e The Roslin Institute and R(D)SVS, University of Edinburgh, Edinburgh, UK; f Centre for Genomic and Experimental Medicine, Institute of Genetics and Molecular Medicine, University of Edinburgh, Edinburgh, UK; g Royal Hospital for Sick Children, Edinburgh, UK; h Nuffield Division of Clinical Laboratory Sciences, Radcliffe Department of Medicine, University of Oxford, Oxford, UK; i Gene Therapy Program, Department of Pathology and Laboratory Medicine, University of Pennsylvania, Philadelphia, PA, USA; j Genzyme, a Sanofi Company, Framingham, MA, USA; k NHS Blood and Transplant, Bristol, UK; l University of Miami, Miami, FL, USA

## Abstract

**Background:**

Lung delivery of plasmid DNA encoding the *CFTR* gene complexed with a cationic liposome is a potential treatment option for patients with cystic fibrosis. We aimed to assess the efficacy of non-viral *CFTR* gene therapy in patients with cystic fibrosis.

**Methods:**

We did this randomised, double-blind, placebo-controlled, phase 2b trial in two cystic fibrosis centres with patients recruited from 18 sites in the UK. Patients (aged ≥12 years) with a forced expiratory volume in 1 s (FEV_1_) of 50–90% predicted and any combination of *CFTR* mutations, were randomly assigned, via a computer-based randomisation system, to receive 5 mL of either nebulised pGM169/GL67A gene–liposome complex or 0·9% saline (placebo) every 28 days (plus or minus 5 days) for 1 year. Randomisation was stratified by % predicted FEV_1_ (<70 *vs* ≥70%), age (<18 *vs* ≥18 years), inclusion in the mechanistic substudy, and dosing site (London or Edinburgh). Participants and investigators were masked to treatment allocation. The primary endpoint was the relative change in % predicted FEV_1_. The primary analysis was per protocol. This trial is registered with ClinicalTrials.gov, number NCT01621867.

**Findings:**

Between June 12, 2012, and June 24, 2013, we randomly assigned 140 patients to receive placebo (n=62) or pGM169/GL67A (n=78), of whom 116 (83%) patients comprised the per-protocol population. We noted a significant, albeit modest, treatment effect in the pGM169/GL67A group versus placebo at 12 months' follow-up (3·7%, 95% CI 0·1–7·3; p=0·046). This outcome was associated with a stabilisation of lung function in the pGM169/GL67A group compared with a decline in the placebo group. We recorded no significant difference in treatment-attributable adverse events between groups.

**Interpretation:**

Monthly application of the pGM169/GL67A gene therapy formulation was associated with a significant, albeit modest, benefit in FEV_1_ compared with placebo at 1 year, indicating a stabilisation of lung function in the treatment group. Further improvements in efficacy and consistency of response to the current formulation are needed before gene therapy is suitable for clinical care; however, our findings should also encourage the rapid introduction of more potent gene transfer vectors into early phase trials.

**Funding:**

Medical Research Council/National Institute for Health Research Efficacy and Mechanism Evaluation Programme.

## Introduction

Cystic fibrosis has been a target for gene therapy since the *CFTR* gene was cloned in 1989.^1^ Lung disease is the main cause of morbidity and mortality in individuals with cystic fibrosis, with a median age at death of 29 years (95% CI 27–31).^2^ Early expectations of a rapid breakthrough were based on supposed ease of access to the target respiratory epithelium via inhaled aerosols. These hopes were tempered by the subsequent realisation that the airways are well defended, in keeping with their predominant function as conducting passages, rather than absorptive surfaces.

**Research in context**

**Evidence before this study**

We searched PubMed between June 1, 1992, and March 1, 2015, for studies published that included the terms “non-viral, gene therapy, cystic fibrosis” or “liposome, gene therapy, cystic fibrosis”.

**Added value of this study**

We report the first trial of non-viral *CFTR* gene therapy for patients with cystic fibrosis that is powered to detect clinically relevant pulmonary changes. Our study has progressed this field of research from phase 1 and 2a studies showing changes in molecular surrogates of CFTR function, to a phase 2b setting assessing changes in lung function in patients with a broad range of *CFTR* mutations. Additionally, our study shows that monthly repeated application of non-viral gene therapy can be safely administered to the lungs over a 1 year period.

**Implications of all the evidence**

By providing the first proof of concept that non-viral gene therapy can beneficially affect lung function, follow-up studies can assess optimum dose, dosing interval, and patient stratification at trial entry. Our findings are likely to catalyse earlier translation of more efficient vectors into first-in-man trials.

Various vectors for delivery of the *CFTR* gene into respiratory epithelial cells have been assessed. Viral approaches, including adenoviruses, adeno-associated viruses, and retroviruses, have faltered because of inefficient transduction from the luminal surface and immune responses restricting the efficacy of repeated application.^3^ As such, research from the UK Cystic Fibrosis Gene Therapy Consortium has initially focused on non-viral vectors. Formulation and delivery of plasmid DNA–liposome complexes have been refined in a large series of preclinical studies,^4^, ^5^ and safety,^6^, ^7^ molecular efficacy, and practical doses have been assessed in several phase 1 and 2a studies in patients with cystic fibrosis.^1^, ^3^ We did this study to assess the clinical efficacy of the non-viral *CFTR* gene–liposome complex pGM169/GL67A^8^ after repeated delivery to the airways.

## Methods

### Study design and participants

We did this randomised, double-blind, placebo-controlled, phase 2b trial in two cystic fibrosis centres with patients recruited from 18 sites in the UK. Eligible participants had diagnosed cystic fibrosis, were aged 12 years or older, had a forced expiratory volume in 1 s (FEV_1_) of 50–90% predicted, and had any combination of *CFTR* mutations.

The protocol was approved by the National Research Ethics Committee and the local Research Committees at the two dosing sites and the 16 other referral centres. Each patient, or a parent, provided written informed consent, and children provided assent.

### Randomisation and masking

We randomly assigned patients (1:1), via a computer-based randomisation system, to receive nebulised pGM169/GL67A or 0·9% saline (placebo). Randomisation was stratified by % predicted FEV_1_ (<70 *vs* ≥70%), age (<18 *vs* ≥18 years), inclusion in the mechanistic substudy, and dosing site (London or Edinburgh). Participants in the mechanistic substudy were randomly assigned (2:1) to receive nebulised pGM169/GL67A or placebo, and could participate as part of either a nasal or bronchoscopy group, or both. Participants and investigators were masked to treatment allocation, with the randomisation code known only by pharmacy staff at the two dosing sites.

### Procedures

Patients received 5 mL of either 0·9% saline or pGM169/GL67A complex nebulised through a Trudell AeroEclipse II device (Trudell Medical International, London, ON, Canada) at 28 day intervals (plus or minus 5 days) for 12 months. Each 5 mL dose of pGM169/GL67A contained 13·3 mg of plasmid DNA and 75 mg of the GL67A lipid mixture. Routine treatments were continued throughout the study, except for DNase, which was withheld for 24 h before and after dosing. In addition to the nebulised dose, patients in the nasal group of the mechanistic substudy received 2 mL of placebo or pGM169/GL67A divided between nasal cavities via a nasal spray device at the time of each lung dose. Patients in the bronchoscopy group followed the standard protocol, but also underwent a bronchoscopy under general anaesthesia before the first dose and 28 days (plus or minus 5 days) after the final dose.

### Outcomes

The primary efficacy endpoint was the relative change in % predicted FEV_1_, calculated from the mean of two baseline values (at screening and before dosing on day of the first dose) to the mean of two values (2 and 4 weeks after last dose) at study completion. Secondary outcomes included additional measurements of lung function, CT scans, and Cystic Fibrosis Questionnaire-Revised (CFQ-R) scores.^9^ Exploratory endpoints included exercise testing, activity monitoring, and sputum inflammatory markers. Mechanistic endpoints were nasal or bronchial vector-specific DNA, mRNA, and electrophysiological assessment of CFTR function. We did extensive safety assessments.

### Statistical analysis

The statistical analyses were prespecified in a statistical analysis plan. With use of pilot data, we estimated the standard deviation of the relative change in % predicted FEV_1_ in the target cystic fibrosis population to be 10% over 12 months. A total sample size of 120 assessable patients would provide 90% power to detect a 6% difference between groups in the mean change from baseline at a two-sided 5% significance level. This power calculation was conservative because covariate adjustment can be expected to increase statistical power. We did analyses in the per-protocol population (primary analysis), predefined as participants who received at least nine doses of pGM169/GL67A or placebo, and in the intention-to-treat population, who received at least one dose of pGM169/GL67A or placebo.

We compared outcomes between groups with an ANCOVA model, with inclusion of the relevant baseline value, treatment allocation, and stratification factors (baseline predicted FEV_1_, age, dosing site, inclusion in substudy). Results are reported as adjusted mean differences with corresponding 95% CIs. We assessed subgroup effects by including the relevant interaction term in the ANCOVA model. To allow results from different endpoints to be plotted on a common scale, the estimated treatment effects were standardised and presented as multiples of the underlying SD. No adjustment was made to the p values to allow for multiplicity because the secondary endpoints were supportive and the corresponding p values were interpreted conservatively. We assessed bronchial and nasal biomarkers with a Mann–Whitney *U* test. A two-sided p value less than 0·05 was considered statistically significant.

The trial was overseen by an independent Data Monitoring and Ethics Committee and a Trial Steering Committee. This trial is registered with ClinicalTrials.gov, number NCT01621867.

### Role of the funding source

The funder of the study had no role in study design, data collection, data analysis, data interpretation, or writing of the report. The corresponding author had full access to all the data in the study and had final responsibility for the decision to submit for publication.

## Results

**Figure 1:**
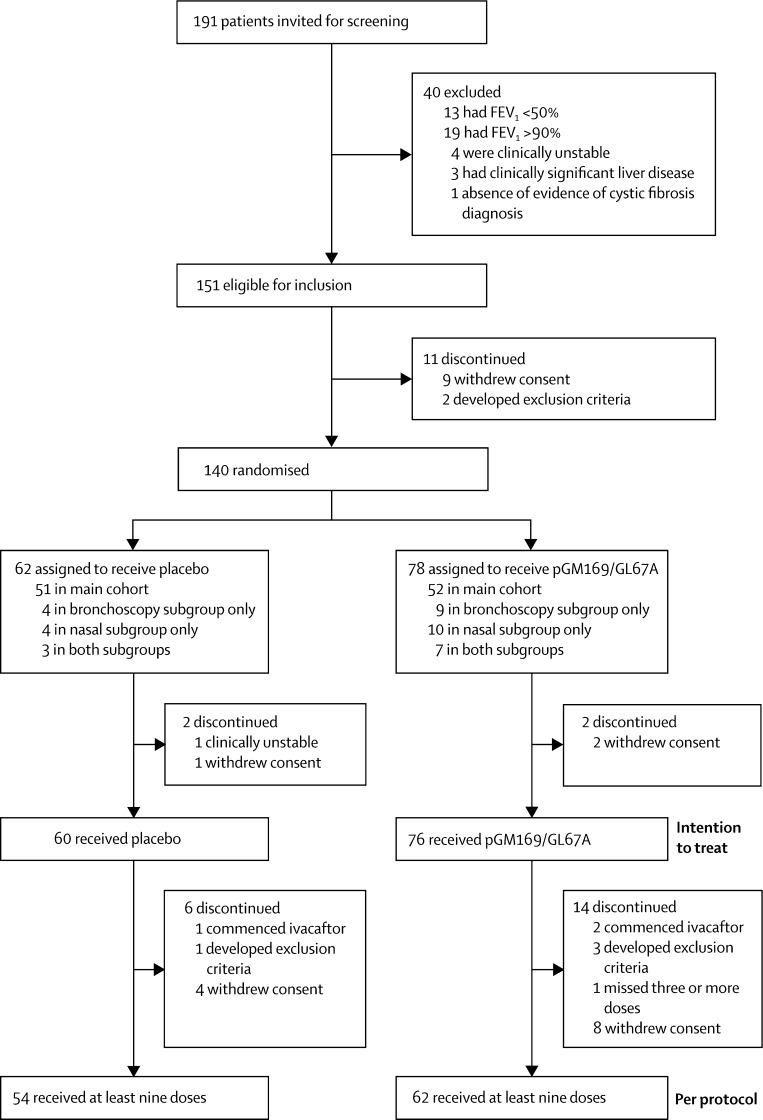
Trial profile Numbers of patients in the intention-to-treat population are unequal because of the 2:1 allocation in the mechanistic substudy. FEV_1_=forced expiratory volume in 1 s.

Figure 1 shows the trial profile. Between June 12, 2012, and June 24, 2013, we randomly assigned 140 patients to receive placebo (n=62) or pGM169/GL67A (n=78), of whom 136 (97%) patients comprised the intention-to-treat population and 116 (83%) patients comprised the per-protocol population (figure 1). Reasons for discontinuation in the intention-to-treat population were similar between groups (appendix). Baseline characteristics were similar between the two groups (table 1). Unless indicated otherwise, all subsequent details relate to the per-protocol population.

**Figure 2:**
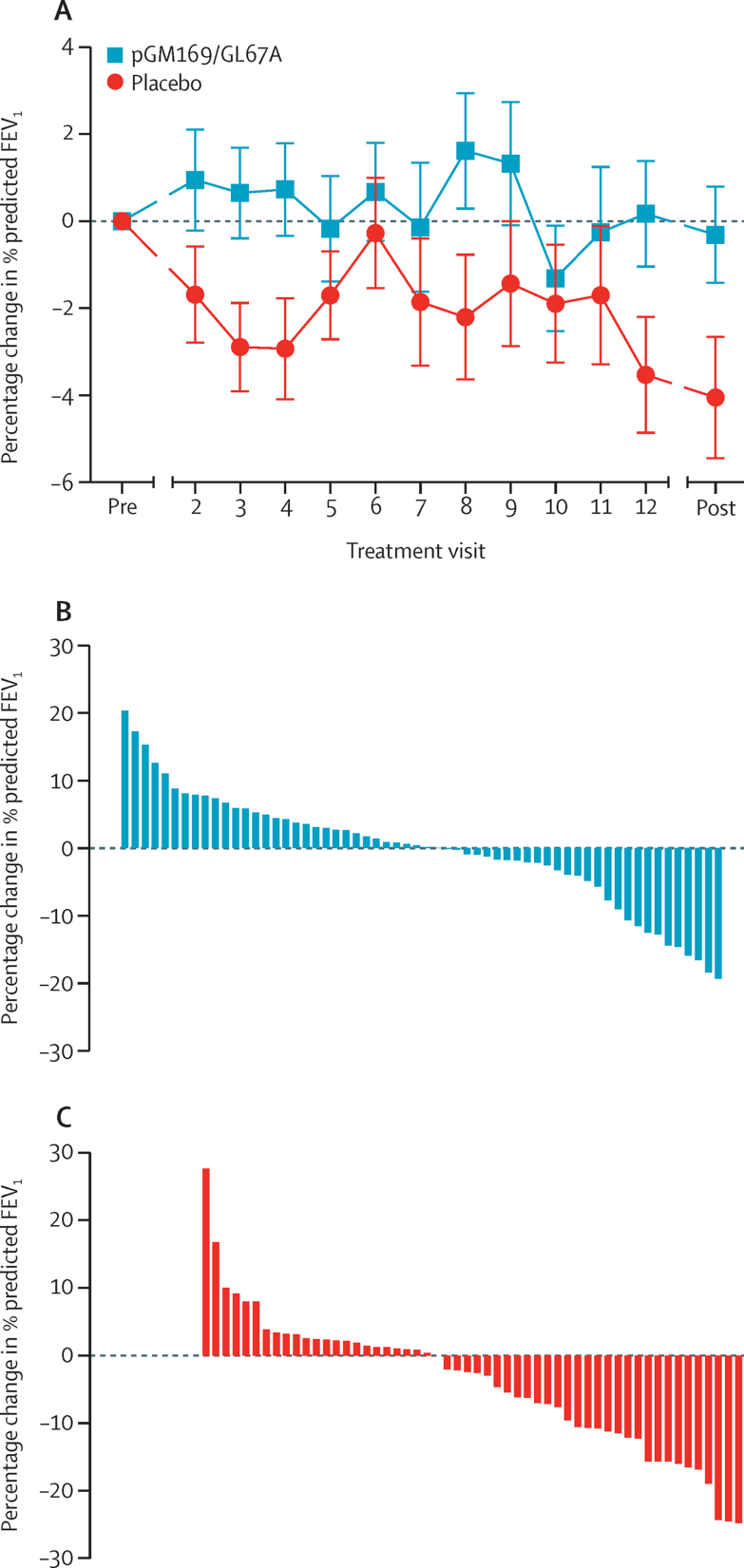
Timecourse of the primary outcome response to either placebo or pGM169/GL67A (A) and the individual patient responses in the pGM169/GL67A (B) and placebo (C) groups Error bars in panel A show the standard error of the mean. Primary outcome measurements were taken at each treatment visit before administration of study drugs. Pre and post values indicate the mean of two measurements at the respective timepoints. Positive values in panels B and C show an improvement. FEV_1_=forced expiratory volume in 1 s.

114 (98%) patients had paired pre-treatment and post-treatment measurements of % predicted FEV_1_. Of the two patients (both in the placebo group) who did not have paired measurements, one patient could not do the test because of a surgery-related pneumothorax and one withdrew because of time commitments and was unavailable for follow-up measurements. We recorded a significant ANCOVA-adjusted treatment effect in the pGM169/GL67A group versus placebo at 12 months' follow-up (3·7%, 95% CI 0·1–7·3; p=0·046; figure 2) The relative changes within each of the individual groups were −4·0% (95% CI −6·6 to −1·4) in the placebo group and −0·4% (−2·8 to 2·1) in the pGM169/GL67A group (figure 2). Post-hoc analysis showed that 21 (18%) patients (n=6 in the placebo group and n=15 in the pGM169/GL67A group) had an improvement in % predicted FEV_1_ of 5% or more of their individual baseline values. For comparison, the treatment effect in patients in the intention-to-treat population who had spirometry measurements both before dosing and within the protocol-defined window after their final dose (n=56 in the placebo group and n=65 in the pGM169/GL67A group) was 3·6% (95% CI 0·2–7·0; p=0·039), with the 20 patients included in the intention-to-treat, but not per-protocol, analysis, receiving a mean of 3·7 doses (SD 1·9).

**Figure 3:**
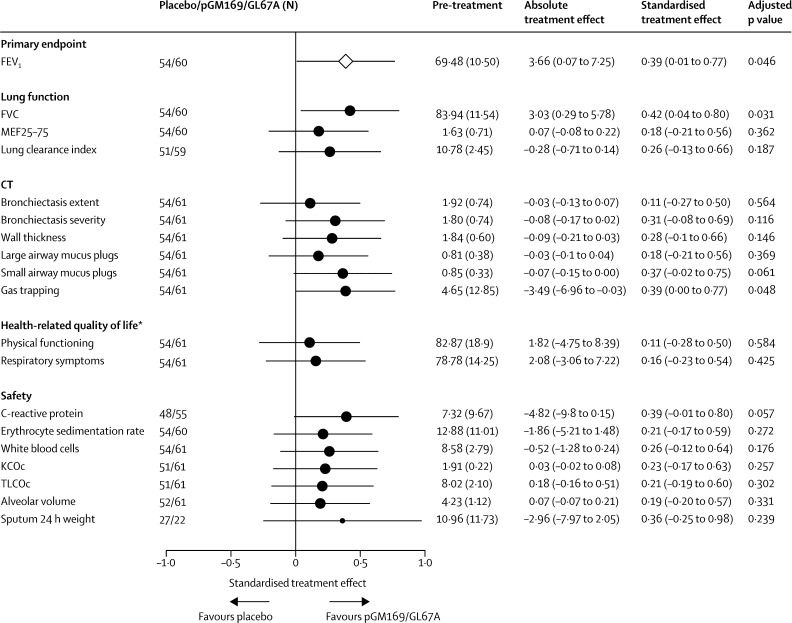
Forest plot showing secondary outcome responses to placebo or pGM169/GL67A Data are mean (SD) or mean (95% CI), unless otherwise indicated. The size of the circles is proportional to the number of patients represented and the error bars show 95% CIs. Values shown for FEV_1_ are the relative change in the % predicted FEV_1_. To allow results from different endpoints to be plotted on a common scale, the estimated treatment effects were standardised to be presented as multiples of the underlying SD (standardised treatment effect). FEV_1_=forced expiratory volume in 1 s. MEF25–75=mid-expiratory flow between 25% and 75% of FVC. KCOc=diffusion capacity of the alveolar capillary membrane, corrected for haemoglobin concentrations. TLCOc=transfer factor of the lung for carbon monoxide, corrected for haemoglobin concentrations. *Refers to scores from the Cystic Fibrosis Questionnaire-Revised.

Figure 3 summarises changes in a range of secondary outcomes. The treatment effect was significant for FVC (p=0·031; appendix) and CT gas trapping (p=0·048), but not for other measures of lung function, imaging, and quality of life (figure 3). We assessed whether a responder subgroup could be identified; the appendix summarises the prespecified subgroups. We noted no significant differences in the primary outcome treatment effect with respect to sex, age, *CFTR* mutation (phe508del homozygous *vs* other), *Pseudomonas* colonisation, predominant smaller or larger airway disease on CT at presentation, concurrent drugs, or treatment-associated adverse events (appendix). Although some subgroups had larger treatment effects than others, these results were typically due to a greater decline in FEV_1_ in the placebo group, rather than to any difference of effect in the pGM169/GL67A group (appendix). Stratification by baseline % predicted FEV_1_ suggested a difference, albeit non-significant, in treatment effect between patients with more severe disease (FEV_1_ 49·6–69·2% predicted), who had a treatment effect of 6·4% (95% CI 0·8–12·1), and those with less severe disease (69·6–89·9% predicted), who had a treatment effect of 0·2% (−4·6 to 4·9; p_interaction_=0·065; appendix). In patients with more severe disease, post-trial and pre-trial changes in both the placebo group (−4·9%) and the pGM169/GL67A group (1·5%) contributed to the treatment effect. Secondary outcomes showed a similar trend favouring the more severe category (appendix).

Patients in both treatment groups received a median of three (IQR one to five) courses of oral or intravenous antibiotics during the trial. Specifically, we assessed co-administered antibiotics during the critical analysis period from dose 11 to the end of the trial. Numbers of patients receiving any additional antibiotics were 26 (48%) in the placebo group and 30 (51%) in the pGM169/GL67A group (χ^2^ p=0·774). Thus, the observed FEV_1_ treatment effect was considered to be independent of concurrent antibiotic courses.

**Figure 4:**
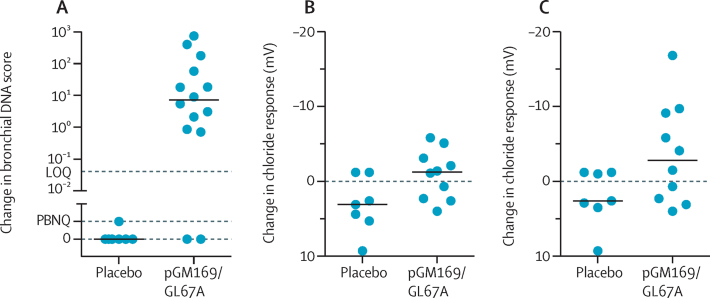
Assessment of DNA from bronchial brushings in the placebo (n=7) and pGM169/GL67A (n=14) subgroups (A) and the response of the bronchial epithelium to perfusion with a zero chloride solution containing isoprenaline 10 μM (B, C) Horizontal bars show median values. Each circle in panel A represents an individual patient. Each symbol in panels B and C shows the change in response from trial start to finish for the relevant treatment in an individual patient. Of the 16 participants in the bronchoscopy subgroup, 15 individuals had post-dose bronchoscopies, of whom 14 individuals generated samples for DNA and mRNA molecular analysis. The plotted value in panel B is the mean of all interpretable recordings (range 1–3), and in panel C is the most negative value obtained from all interpretable recordings, at each timepoint for that patient. A more negative value is in the non-cystic fibrosis direction. LOQ=limit of quantification, PBNQ=positive but not quantifiable.

No clinically relevant pattern of changes could be distinguished in the exploratory outcomes of activity and exercise monitoring and serum and sputum inflammatory markers (appendix). In the bronchoscopy group of the substudy, vector-specific DNA increased in 12 (86%) of 14 patients in the pGM169/GL67A group and was below the limit of quantification in all (n=7) placebo samples (p=0·001; figure 4A); vector-specific mRNA was below the level of sensitivity in both groups (appendix). Changes in basal post-trial and pre-trial potential difference values did not differ significantly in either group (appendix). Figure 4B shows bronchial chloride responses using the mean of all interpretable tracings for each patient; a negative value indicates a change in the non-cystic fibrosis direction. Patients in the placebo group (n=7) had a median change (post-trial minus pre-trial) of 3·1 mV (range 9·3 to −1·2) and those in the pGM169/GL67A group (n=10) had a change of −1·3 mV (4·0 to −5·8; p=0·032; figure 4B). Five (50%) of ten patients in the pGM169/GL67A group had values that were more negative than the largest response in the placebo group (figure 4). In the same analysis with only the most negative value recorded for each patient at any timepoint, patients in the placebo group had a median post-trial minus pre-trial change of 2·6 mV (range 9·3 to −1·2) and those in the pGM169/GL67A group had a change of −2·8 mV (4·0 to −16·8 mV; p=0·088; figure 4C). Six (60%) patients in the pGM169/GL67A group had values that were more negative than the largest response in the placebo group (figure 4). The appendix shows absolute bronchial potential difference values.

In patients in the nasal group of the substudy, vector-specific DNA increased in all the 17 patients given pGM169/GL67A. Despite apparent pGM169 contamination in some samples, the change in pGM169 concentrations differed significantly between the groups (appendix); no vector-specific mRNA was quantifiable in either group. We noted no significant changes in the baseline, zero chloride, or isoprenaline responses (appendix). Four (29%) of 14 pGM169/GL67A patients had mean post-trial minus pre-trial treatment responses (ranging from −3·4 mV to −7·0 mV) that were more negative than the largest response in the placebo group (n=6; appendix). The appendix shows absolute nasal potential difference values.

All patients had adverse events, with no significant difference between groups for either total events or within the nine predefined adverse event categories (table 2). One patient in the placebo group and one patient in the pGM169/GL67A group discontinued study treatment because of adverse events (fatigue and increased respiratory symptoms and flu-like symptoms, respectively). We recorded six serious adverse events, all in the pGM169/GL67A group (appendix). Neither the Data Monitoring and Ethics Committee nor the Trial Steering Committee regarded any serious adverse event as related to study drug; however, one event was considered to be possibly related to a trial procedure (bronchoscopy). We noted no clinically relevant changes in haematology, biochemistry, conversion of anti-CFTR T cells, anti-DNA antibodies, histology, or lipid staining (appendix) and no patients died during the study.

## Discussion

We report the first trial of non-viral based gene therapy for cystic fibrosis, powered to detect clinically relevant pulmonary changes. After monthly dosing for 1 year, we recorded evidence of a beneficial effect of gene therapy versus placebo on FEV_1_. No effect of sex, age, or whether patients were homozygous for the most common F508del *CFTR* mutation could be detected. No clinically important adverse events attributable to treatment with pGM169/GL67A were reported.

Although these findings are encouraging, they should be put into perspective. We noted a stabilisation of FEV_1_ in the pGM169/GL67A group rather than an improvement. This stabilisation took place over a 1 year period and further work will be needed to see if this effect is maintained. The reduction in FEV_1_ in the placebo group was within the range reported in some other prospective trials^10^, ^11^, ^12^ and is consistent with a median survival of 29 years, but is greater than would be expected from registry data.^2^ Three factors are likely to have influenced this difference. First, the requirement for clinical stability at trial entry meant that patients might have been at their optimum respiratory health at this stage. Second, the enthusiasm of patients to enter the trial, accompanied by a focus on self-care, might have resulted in short-term improvements in lung function during the recruitment period. Both factors are likely to lead to a subsequent decline in lung function as patients regress to their mean values. Third, we included all available data, whether from stable patients or those with exacerbations, by contrast with registry data, which focuses on measurements obtained at annual review. Stabilisation of lung disease in itself is a worthwhile aim and we would caution against the bar being set too high for novel therapeutics in cystic fibrosis populations with an unselected range of mutations. The large response to ivacaftor in patients with class III mutations takes place in the context of correctly localised CFTR protein. By contrast, much smaller improvements in lung function were shown in the ivacaftor–lumacaftor trial for the most common mutation (phe508del) in which the CFTR protein is misfolded and mislocalised.^13^

The response in our study was heterogeneous, with apparent responders and non-responders. The data suggest that an approximate doubling of treatment effect was achieved in patients with more severe disease stratified by baseline FEV_1_, supported by trends in other clinically relevant secondary measures. A larger trial with a stratified trial entry design, powered to assess subgroups, and that addresses the mechanisms of response heterogeneity, will be important to verify or refute these data. This differential response could relate to the dose deposited in the airways; in patients with lower baseline FEV_1_ the relatively more obstructed smaller airways result in a larger proportion of the 5 mL dose being deposited in the larger airways. In pre-trial studies we assessed airway deposition in patients with cystic fibrosis with varying FEV_1_ severity with technetium-99m labelled human serum albumin of similar droplet size (3–4 μm, using a different nebuliser system) to the pGM169/GL67A formulation. Bronchial airway (generations 2–8) fractional deposition was 2·9% of delivered dose (standard error of the mean [SEM] 0·2; n=33) in patients with 70–90% predicted FEV_1_ and roughly twice as great (6·0%, SEM 1·0; n=23) in those with 50–70% predicted FEV_1_. An additional contributory factor to this enhanced efficacy might be the increased mitotic rate of more severely affected tissues,^14^ which decreases the proportion of time that the nuclear membrane is intact, the membrane acting as a barrier to plasmid DNA entry to the nucleus.

We cannot rule out that the changes recorded in the present study are the result of a non-specific response to the pGM169/GL67A formulation. The placebo was 0·9% saline rather than a scrambled or *CFTR*-deleted plasmid–liposome complex. We selected 0·9% saline partly on the basis of pragmatic financial considerations, but mainly for ethical considerations, not wishing to expose patients with cystic fibrosis to first-in-man repeated pulmonary dosing of an untested product that might direct the expression of an immunologically active peptide or novel non-coding RNA molecule with deleterious biological functions. Furthermore, we wanted to compare progression on therapy with the natural history of the disease. In terms of alternative explanations for the effects we noted, we know of no evidence that monthly nebulisation of 0·9% saline is deleterious to lung function, nor that liposome alone produces physiological improvements in either patients without,^15^ or those with^16^ cystic fibrosis. Delivery of non-CFTR encoding plasmid DNAs to the human airways has not been associated with a gain in CFTR chloride-channel function, nor improvement in any cystic fibrosis-related assay,^17^, ^18^ and plasmid DNA is generally associated with pro-inflammatory, rather than non-specific, beneficial effects.^19^ We did not identify any pathophysiological changes in the airways, such as inflammation or remodelling, nor any changes in bacterial species that might otherwise explain the outcomes. Nevertheless, we cannot exclude that DNA–liposome complexes augment host defences, stimulate mucus clearance, or enhance bacterial killing to an extent undetectable on semi-quantitative routine culture.

Results showing more robust changes in molecular CFTR surrogates would have been reassuring. Despite extensive optimisation of quantitative realtime-PCR assays, the pGM169-derived mRNA assay has poor sensitivity and is adversely affected by the inclusion of high levels of total RNA or modest concentrations of pGM169 plasmid DNA. In ovine studies we have shown that a 20 mL nebulised dose of pGM169/GL67A, four times that used in the present trial, is the lower threshold for reproducible detection of mRNA with this assay in airway tissue samples (unpublished).^6^ Thus, our inability to detect pGM169-derived mRNA after delivery of 5 mL of pGM169/GL67A to the human airways, although disappointing, was not surprising. In human tissues, we have noted the low sensitivity of assays assessing vector-specific mRNA from human samples in vivo,^16^, ^20^, ^21^ and have noted the greater sensitivity of detection of electrophysiological changes, consistent with findings in this study.^17^, ^18^, ^22^

The ratio of area sampled to area dosed is small. Although we recorded significant chloride secretory changes in the bronchial, but not the nasal, epithelium, we caution against placing undue weight on either observation. The size of the groups in the mechanistic substudy was limited by both the practicality of the procedures and the acceptability to patients of the additional invasive tests, leading to low statistical power for these measures. We would instead conclude that modest variable changes can be shown with currently available assays that remain insufficiently sensitive to detect changes in low levels of CFTR function when assessed in vivo in humans; further optimisation in these or other assays is needed.

Although we are encouraged by the first demonstration of a significant beneficial effect in lung function compared with placebo associated with gene therapy in patients with cystic fibrosis, the mean difference was modest, only recorded in some individuals, and at the lower end of the range of results seen in clinical trials which result in changes in patient-related care.^23^, ^24^ We did not formally assess infective exacerbations in view of the fairly small patient numbers in our study, but use of antibiotic courses as a surrogate identified no obvious treatment advantage. The treatment effect is consistent with a clinically meaningful benefit from the perspective of the European Medicine Agency;^25^ however, further improvements in efficacy and consistency of response to the current formulation, or its combination with CFTR potentiators, are needed before gene therapy is suitable for clinical practice. Furthermore, our findings should encourage the rapid introduction of more potent gene transfer vectors into early phase trials, now that much of the groundwork has been established.

The data reported here provide the first proof of concept that repeated administration of non-viral *CFTR* gene therapy can safely change clinically relevant parameters, providing another step along the path of translational cystic fibrosis gene therapy.

For the **UK Cystic Fibrosis Gene Therapy Consortium** see http://www.cfgenetherapy.org.uk

**This online publication has been corrected. The corrected version first appeared at thelancet.com/respiratory on September 7, 2015**

## Figures and Tables

**Table 1:** Baseline and demographic characteristics

		**Placebo group (n=54)**	**pGM169/GL67A group (n=62)**
Age (years)		26·0 (13·0)	23·6 (10·8)
	<18 years old	17 (31%)	23 (37%)
	≥18 years old	37 (69%)	39 (63%)
Sex			
	Female	25 (46%)	31 (50%)
	Male	29 (54%)	31 (50%)
Centre distribution number			
	Edinburgh	24 (44%)	22 (35%)
	London	30 (56%)	40 (65%)
Height (cm)		165·0 (10·6)	163·6 (10·9)
Weight (kg)		61·6 (15·6)	61·0 (15·7)
FEV_1_ (% predicted)		69·0 (9·9)	69·9 (11·1)
Body-mass index (kg/m^2^)		22·4 (4·4)	22·4 (4·5)
Mutation class			
	Phe508del/Phe508del	26 (48%)	31 (50%)
	Phe508del/class 1–6	22 (41%)	23 (37%)
	Not Phe508del/class 1	1 (2%)	3 (5%)
	Heterozygous/homozygous class 3–6	2 (4%)	2 (3%)
	Phe508del/unknown class	3 (6%)	3 (5%)

Data are mean (SD) or n (%), unless otherwise indicated.

**Table 2:** Adverse events

	**Placebo group (n=54)**	**pGM169/GL67A group (n=62)**
Lower airway respiratory symptoms	7·9	9·0
Gastrointestinal symptoms	2·1	1·8
Fever or flu-like symptoms	1·1	1·4
Headache	1·2	1·1
Upper airway symptoms	2·3	3·4
Elevated liver function tests	0·3	0·4
Haematuria	0·2	0·2
Isolated raised inflammatory markers	0·8	0·7
Other	3·2	3·3
Total	19·1	21·2

Data are mean number of times the respective symptom was experienced by each patient during the trial. Values were calculated by dividing the total number of the relevant adverse event by the total number of relevant patients in that group.
