# Corrections

**DOI:** 10.1016/S2213-2600(15)00314-8

**Published:** 2015-09

*Alton EWFW, Armstrong DK, Ashby D, et al, on behalf of the UK Cystic Fibrosis Gene Therapy Consortium. Repeated nebulisation of non-viral CFTR gene therapy in patients with cystic fibrosis: a randomised, double-blind, placebo-controlled, phase 2b trial.* Lancet Respir Med 2015; *published online July 3. http://dx.doi.org/10.1016/S2213-2600(15)00245-3—*In this Article, Catherine McKenny (Oxford Churchill) was missing from the Acknowledgments, as was reference to financial support from NHS Research Scotland, through the Edinburgh Clinical Research Facility. This correction has been made to the online version as of Sept 7, 2015, and the printed version is correct.

