# Supplementary material for: Repeated nebulisation of non-viral CFTR gene therapy in patients with cystic fibrosis: a randomised, double-blind, placebo-controlled, phase 2b trial
Source: Lancet Respir Med. 2015 Sep;3(9):684–91. doi: 10.1016/S2213-2600(15)00245-3 (PMC4673100; doi:10.1016/S2213-2600(15)00245-3)
Supplement: Supplementary appendix [file mmc1.pdf]

THE LANCET Respiratory Medicine

Supplementary appendix

This appendix formed part of the original submission and has been peer reviewed. We post it as supplied by the authors.

Supplement to: Alton EWFW, Armstrong DK, Ashby D, et al, on behalf of the UK Cystic Fibrosis Gene Therapy Consortium. Repeated nebulisation of non-viral CFTR gene therapy in patients with cystic fibrosis: a randomised, double-blind, placebo-controlled, phase 2b trial. *Lancet Respir Med* 2015; published online July 3. http://dx.doi.org/10.1016/S2213-2600(15)00245-3.

Supplementary Appendix

Repeated nebulisation of non-viral *CFTR* gene therapy in patients with cystic fibrosis: a randomised, double-blind, placebo-controlled, phase 2b trial

CONTENTS

METHODS
1. Randomization
2. Protocol
3. Schedule of visits and investigations
4. Therapeutic formulation
5. Dose administration
   a) Nebulisation
   b) Nasal dosing
   c) Concurrent medications
6. Biochemistry, haematology and serum/sputum/urine inflammatory markers
7. Cell counts and lipid staining of sputum cells
8. Immunological markers
9. Histology
10. Lung physiology
    a) Spirometry
    b) Lung clearance index
    c) Gas transfer
11. Computerised tomography lung scans
12. VO_2 max
13. Activity monitoring
14. Bronchoscopic procedures
15. Nasal brushing
16. Quantification of DNA and mRNA
17. Nasal potential difference
18. Statistical Analysis Plan

RESULTS
1. Reasons for discontinuation, once subjects were randomized, prior to dose 1
2. Reasons for discontinuation once subjects were enrolled in the ITT population
3. Interim safety analysis
4. ITT intra-group analysis
5. Sputum microbiology
6. Subgroup analysis
7. CFTR molecular outcomes in nasal arm of sub-study
8. CFTR molecular outcomes in bronchial arm of sub-study
9. Adverse events

REFERENCES

SUPPLEMENTARY FIGURE & TABLE LEGENDS

SUPPLEMENTARY FIGURES & TABLES

Figures S1-S10

Tables S1-S4

METHODS

1. **Randomisation**
   Randomisation was performed, following a successful screening visit, via the on-line InForm database system (Oracle Health Sciences, Reading, UK). All data required for randomisation and stratification were entered by a member of the trial team which led to the generation of a unique patient number, corresponding to a blinded, randomised arm of the trial. In the event of computer system failure, a pre-arranged manual randomisation method was available from the InForm team. The unique patient number was entered onto the subject's prescription sheet and submitted to the trial pharmacists who had access to the unblinding code and prepared the Active or Placebo product as appropriate.

2. **Protocol**
   The protocol was designed by the GTC and the trial sponsor was Imperial College, London, UK. The data were analysed by both the Imperial College Clinical Trials Unit and the investigators. All senior investigators prepared the initial drafts; all authors participated in the subsequent revision and vouch for the accuracy of the data, and for fidelity of the study to the protocol.

3. **Schedule of visits and investigations**
   Although various exploratory assays and other measurements were performed at interim time-points, the analysis of the primary and secondary endpoints analysis was restricted to samples and measurements taken prior to Dose 1 and after the last dose. The complete Schedule is shown below.

Summary of Study Assessments

Week								0		4		8		12	16	20	24	28	32	36	40	44	46	48	49
Visit name	E&C	Intro	Screen	nPD1[E]	nPD2[E]	nPD3[E]	Bronch[E]	dose1	D2a	dose2	D2b	dose3	D2c	dose4	dose5	dose6	dose7	dose8	dose9	dose10	dose11	dose12	F/U1	F/U2	Bronch
**CONSENT/ HISTORY/ EXAMINATION**																									
Informed Consent	X[A]	X[A]	X[A]				X[H]																		
Medical History	X	X	X				X	X	X	X	X	X	X	X	X	X	X	X	X	X	X	X	X	X	X
Physical Examination	X	X	X				X	X	X	X	X	X	X	X	X	X	X	X	X	X	X	X	X	X	X
Quality of Life Questionnaire (CFQ-UK)			X											X			X			X			X	X	
Vital signs [1]	X	X	X				X	X	X	X	X	X	X	X	X	X	X	X	X	X	X	X	X	X	X
Pulse oximetry	X	X	X				X	X	X	X	X	X	X	X	X	X	X	X	X	X	X	X	X	X	X
Prior medications	X[A]	X[A]	X[A]																						
Concomitant medications			X				X	X		X		X		X	X	X	X	X	X	X	X	X	X	X	X
Check diary comments and reissue diary								X		X		X		X	X	X	X	X	X	X	X	X	X	X	X
Adverse events							X	X	X	X	X	X	X	X	X	X	X	X	X	X	X	X	X	X	X
Download PiKo-6 device			X[K]				X	X		X		X		X	X	X	X	X	X	X	X	X	X	X	X
Sweat test [R]	X [R]	X [R]	X [R]																						
**SAMPLES[J]**																									
Blood sampling	X	X	X					X	X	X	X	X	X	X	X	X	X	X	X	X	X	X	X	X	
Urine sampling	X	X	X					X	X	X	X	X	X	X	X	X	X	X	X	X	X	X	X	X	
Sputum[Q]		X[LP]	X[LP]					X[L]	X	X[N]	X		X			X			X				X[P]	X[P]	
Sputum for NTM	X[M]	X[M]	X[M]					X[M]															X[M]	X[M]	
24 hour sputum weight		X	X					X	X	X	X	X	X	X				X					X	X	
**INVESTIGATIONS[J]**																									
Lung Clearance Index		X [L]	X [L]					X [L]						X			X			X			X	X	
Exercise Bike test			X[M]					X[M]						X[M]	X[M]					X				X	
Activity monitor			X													X							X		
CT scan			X											X[F]										X	
Gas transfer			X						X		X		X	X			X							X	
Bronchial blood flow measurement[G]			X											X										X	
Spirometry	X	X	X				X	X	X	X	X	X	X	X	X	X	X	X	X	X	X	X	X	X	X
**DOSING**																									
Randomisation[B]								X																	
Study dose administration								X		X		X		X	X	X	X	X	X	X	X	X			
**NASAL/ BRONCH SUBGROUPS ONLY**																									
Nasal potential difference	X[C]	X[C]	X[C]	X[C]	X[C]	X[C]						X [S]		X[G]		X [S]	X		X [S]	X[G]			X[L]	X[L]	X[L]
Nasal brushing			X[D]			X[D]																		X[M]	X[M]
Bronchoscopy							X																		X
**PRE-DISCHARGE ADMIN**																									
Instructions re home spirometry (PiKo-6)		X	X					X		X		X		X	X	X	X	X	X	X	X	X	X		
Instructions re use of symptom diary		X	X					X		X		X		X	X	X	X	X	X	X	X	X	X		
Schedule next study appointment		X	X					X	X	X	X	X	X	X	X	X	X	X	X	X	X	X	X		

Superscripts

A- first point of contact only
B- to be conducted within week prior to visit
C- 3 occasions only
D- one occasion only with final nPD
E- may occur before Screening
F- limited cut
G- if available; timing may vary
H- bronchoscopy-specific consent only
J- on dosing visits, must be pre-dose
K- Patients who have undergone Intro visit only
L- 2 time points only
M- one time point only
N - for intensively monitored cohort only
P - Can induce at these timepoints; required once pre and once post dosing
Q- if no sputum available, cough swab to be performed
R - one time point pre-dosing if required
S - Optional at day14 post dosing
Subgroups in grey areas:
Intensive cohort, new subjects,
nasal/ bronch cohorts

4. Therapeutic formulation

Reconstitution and preparation of pGM169/GL67A was undertaken by the designated clinical trial pharmacists according to MHRA-approved protocols. Lipid GL67 (gift from Sanofi-Genzyme Corporation, Cambridge, MA, USA) was formulated together with DOPE (Avanti Polar Lipids, Alabaster, AL, USA) and DMPE-PEG5000 (Avanti Polar Lipids) at a molar ratio of 1:2:0·05 (GL67:DOPE:DMPE-PEG5000) to form GL67A as previously described[1] by OctoPlus (Leiden, Netherlands). Freeze-dried GL67A lipid films containing ~150 mg total lipid were rehydrated with 5·3 ml of sterile water for injection immediately prior to formulation to a final concentration of ~28·6 mg/ml. The CpG-free plasmid pGM169 expressing a codon-optimised *CFTR* gene under control of the hCEFI promoter[2] was supplied at ~5·3 mg/ml in sterile water for injection by VGXi Inc (The Woodlands, Texas USA).

pGM169/GL67A complexes were prepared by simultaneous extrusion of equal volumes of the pGM169 plasmid DNA and GL67A lipid mixture through an 8-element divide/merge static mixer using a purpose built LMD-2 pneumatic mixing device[3] operating at a fluidic mixing rate of 2·5 ml/s. The resulting lipid/DNA complexes were delivered to patients within 6 hr of mixing.

Five ml volumes of pGM169 (13·25 mg)/GL67A (75 mg) or Placebo (saline) were placed in AeroEclipse II breath-actuated nebulisers (Trudell Medical Instruments, London, ON, Canada). To avoid unblinding, nebulisers were taped and a tamper-proof seal was attached. Trial medicines were prepared by unblinded trial pharmacists. Clinical trial staff, participants and trial analysts were blind to allocation until database lock. 10 ml volumes were placed in opaque nasal spray devices (GSK parts No. AR5989 30mL bottle/AR9488 30 ml actuator) and the device was primed.

5. Dose administration

a) Nebulisation

Delivery took place in sealed negative-pressure cubicles with external venting to limit spread within the outpatient hospital setting; patients could be observed and communicated with through a glass window. Each 5 ml aliquot was nebulised during cycles of tidal breathing for 3 minutes, following which the nebuliser air supply (8 L/min) was turned off for a 2 minute rest period. Nebulisation continued for 40 mins, predetermined as a period after which delivery was complete. The measured MMAD (mass median aerodynamic diameter) of pGM169/GL67A aerosols using the AeroEclipse II was 3·4 ± 0·1 µm with a fine particle fraction (FPF) of 71·4 ± 1·5%.[4] To confirm that nebulisation does not alter the ability of pGM169/GL67A to generate functional chloride channels, HEK-293T cells were transfected with pGM169/GL67A collected both prior to nebulisation, and at the end of nebulisation (as residual volumes in the nebuliser). Samples were then assessed in an iodide efflux assay, commonly used to assess CFTR function, as previously described.[5] Levels of cAMP-mediated iodide efflux were similar when using pGM169/GL67A before, and after, nebulisation (data not shown).

b) Nasal dosing

The nasal subgroup patients (n=24 ITT, n=20 PP) received a total of 24 actuations of the pGM169/GL67A formulation, in total delivering approximately 2 ml equally divided between the two nostrils, during the 2 min nebulisation rest periods.

c) Concomitant medications

Salbutamol (200-400 µg) was administered via metered dose inhaler and spacer device approximately 20 minutes prior to dosing to minimise any bronchoconstrictive effect. A standard, weight-adjusted, clinical dose of paracetamol was administered within the first 2 hours post-dosing and a second dose given to subjects to take at home 6 hours later to minimise any inflammatory response and prevent unblinding.

6. Biochemistry, haematology, serum/sputum/urine inflammatory markers

Blood samples for electrolytes, renal function, C-reactive protein (CRP), full blood count and coagulation screen were obtained by venepuncture and processed by the clinical laboratories in accordance with usual routine hospital practice. Serum IL-6 was measured on the Beckman Access 2 immunoassay analyser (Beckman Coulter, High Wycombe, Buckinghamshire, UK). Urine was assessed by standard 'dipstick' analysis.

Serum was frozen and stored for subsequent cytokine analysis. An in-house calprotectin ELISA was used (intra-assay coefficient of variation = 5·6%: unpublished observations). Calprotectin antibodies were kind gifts of Erling Sundrehagen, Oslo, Norway. Incubations were carried out in a damp box. Microtitre plates (Dynex Immulux HB) were coated overnight at 4°C with 100 µl anti-calprotectin (mouse anti-human) monoclonal antibody at 2·5 µg/ml in coating buffer (KPL/Insight Biotechnology). Plates were blocked with 1% BSA for 1 h at 37°C and washed four times with 0·05% Tween 20. One hundred µl duplicate samples were added to the plate in 1/60000 dilutions for sputum (in phosphate buffered saline (PBS)) and 1/500 for serum (50% fetal calf serum in PBS). Positive controls of calprotectin (Immunodiagnostik, Oxford Biosystems) were included. Human recombinant calprotectin (Cambridge Biosystems) was used to produce 1·56 - 100 ng/ml standard curves. The samples; anti-calprotectin (chicken anti-human) polyclonal antibody at 1/5000 dilution; 100 µl of Alkaline Phosphatase Conjugated Donkey anti-Chicken IgG (Jackson ImmunoResearch, Suffolk, UK) at 1/5000 dilution were added to wells in three cycles of incubation at room temperature for 1 h on a platform vibrator (450 rpm) followed by washings. Finally, 100 µl Bluephos Phosphatase Substrate System (KPL) was added to each well. Plates were incubated in the dark at 600 rpm until the blue colour developed, and 620 nm absorption read on a Biotek Plate reader. Sample concentrations were calculated using the 5 Parameter Logistic (5PL) nonlinear regression curve fitting model.

Patients were asked to collect sputum in 50 ml Falcon tubes 24 hr prior to a scheduled hospital visit. Tubes were then weighed to determine the amount of sputum produced over a 24 hr period and subsequently discarded. 97 out of the expected 232 samples (42%) were not available due to patients either not spontaneously expectorating sputum or forgetting to undertake the collection.

During trial visits (before dose 1 and after dose 12) sputum was also collected. Prior to dose 1 sputum was expectorated in 72 out 116 (62%) of patients (Active: 37/62 (60%), Placebo: 35/54 (65%). After the last dose sputum was expectorated in 57 out 116 (49%) of patients (Active: 27/62 (44%), Placebo: 30/54 (56%)). Freshly expectorated sputum was stored on ice for a maximum of 2 hr and processed as previously described.[6] In cases where patients did not produce sufficient sputum to perform all sputum assays the following priority for assays was assigned: (1) clinical microbiology (generated from cough swabs if patients were completely non-productive), (2) soluble sputum which generated samples for quantification of IL-8, calprotectin and extracellular DNA as well as sputum cells, (3) sputum solid content. Samples were processed for routine bacterial and fungal culture according to CF-specific clinical SOPs at each time point. In addition, once pre-dosing and at follow-up, sputum (or bronchial aspirate where appropriate) was cultured for non-tuberculous mycobacteria according to standard clinical procedures.

Samples to prepare soluble sputum were available from 130 of 232 samples (56%). Sputum calprotectin was measured as described above. Sputum IL-8 assays were performed using a commercial kit (Human IL-8 Ultra Sensitive Kit, Kit No. KAC1301, Biosource/Invitrogen, CA, USA), following the manufacturer's instructions. Plates were read using a Biotek Plate Reader, and standard curves produced using the 5 Parameter Logistic (5PL) nonlinear regression curve fitting model. Extracellular DNA was quantified in the soluble sputum fraction using Quant-iT PicoGreen dsDNA Assay Kit (Invitrogen, Paisley, UK) according to manufacturer's recommendations. Sputum solid content was determined as previously described.[6] 169 out of the expected 232 samples (63%) were not available due to patients not expectorating sufficient sputum.

7. Cell counts and lipid staining of sputum cells

Cells were isolated from solubilised sputum as described above and approximately 10^5 sputum cells were cytospun (5 min, 500 rpm) onto cytoslides (Thermo Shandon Ltd, Cheshire, UK) (6 slides/subject). Slides were air-dried and fixed for 10 min in 4% Formalin, (Sigma-Aldrich, St Louis, USA) and subsequently stored at -20°C until further use. For total and differential cells counts, cells were fixed for 10 min in cold methanol (Fisher Scientific, Loughborough), dried and stained with May-Grünwald-Giemsa (MGG quick stain, Bio-Optica, Milano, Italy) using routine histological procedures and neutrophils, lymphocytes, macrophages and eosinophils quantified. Cells were counted on random fields until 300 neutrophils were counted. Total and differential cell counting could be performed on 129 of 232 (56%) and 111 of 232 (48%) samples, respectively.

Quantification of intracellular lipid droplets could be performed on 121 of 232 (52%) of samples. Slides were stained with Oil Red O (ORO, Alfa Aesar, Heysham, UK) using a protocol adapted from Lian X. *et al.*[7] Briefly, defrosted slides were washed in distilled water, dried and placed for 3 min in 100% propylene glycol (Sigma-Aldrich, St Louis, USA). Slides were then transferred into pre-warmed (60°C) ORO solution (0·5% w/v in propylene glycol) and stained for 10 minutes in a 60°C oven. Subsequently slides were place into 85%

propylene glycol for 3 min at room temperature and rinsed three times in distilled water, counterstained with Harris Heamatoxylin and mounted in Aquatex mounting medium (VWR International Ltd., UK). Quantification of lipid staining was performed blinded. On each slide three hundred neutrophils, one hundred macrophages and one hundred squamous cells were evaluated for the presence of cytoplasmic lipid droplets. The data are presented as % of lipid containing cells.

8. Immunological markers

Anti-ds (double stranded) DNA antibodies (IgG) were processed by the clinical laboratories using a commercial ELISA dsDNA assay (Phadia 250, ThermoScientific) in accordance with routine hospital practice. 218 of 232 samples (94%) were available for analysis. Peripheral blood mononuclear cells were extracted and human interferon-γ enzyme-linked immunospot assay for the analysis of CFTR-specific T cells was performed as previously described.[8] 170 of 232 samples (73%) were suitable for analysis. In the remaining 27% of samples, cell viability was too low to perform the assay. Cell viability was likely affected by storage of peripheral blood mononuclear cells (PBMC) in liquid nitrogen and shipment of samples from the UK to the USA, where the assays were performed.

9. Histology

Two bronchial biopsies were collected from each patient. One was frozen in isopentane-cooled liquid nitrogen at -196°C and the other immediately fixed in 10% formal saline. The formalin fixed samples were processed using a Tissue Tek Sakura VIP processor and embedded in paraffin wax blocks. Three µm sections were then cut from these blocks for H&E staining. For frozen samples, 6-8 µm sections were cut for haematoxylin and eosin (H&E) and Oil Red O (ORO) staining. For H&E staining, slides were thawed at room temperature for 30 mins, and then placed in Harris' haematoxylin for 1 min. Subsequently, they were differentiated in acid alcohol for 5 secs and left to blue in tap water for 5 min. They were then counterstained with eosin for 30 seconds and cover-slipped using an aqueous mountant.

For ORO staining, frozen sections were thawed and air dried at room temperature for 30 mins. Slides were rinsed in 60% isopropanol then placed in filtered ORO working solution for 15 mins. Subsequently, the slides were taken out of the ORO solution and excess dye was washed off with 60% isopropanol, before rinsing in three changes of distilled water. The sections were counterstained using Harris' haematoxylin for 30 secs in order to visualize the nuclei. The sections were left to blue in running tap water for 5 mins and mounted with a coverslip using Aquatex (an aqueous mountant). H&E slides were scored independently by two blinded pathologists using a semi-quantitative scoring system for goblet cell hyperplasia, basement membrane thickening, presence of chronic inflammatory cells (lymphocytes and plasma cells), neutrophils, eosinophils and seromucinous gland hyperplasia. ORO slides were scored for the presence of lipid-laden macrophages using a semi-quantitative scoring system.

10. Lung physiology

a) Spirometry

Spirometry was performed using an EasyOne® spirometer according to ATS/ERS standards. Conversion of the raw values for FEV_1, FVC and MEF_{25-75} to % predicted values was undertaken according to the Stanojevic reference source.[9]

b) Lung clearance index

Lung clearance index (LCI) was measured using a multiple breath washout technique with a modified Innocor device (Innovision, Odense, DK) as previously described (6). Briefly, 0·2% SF_6 in air was ‘washed in’ to the lungs until equilibrium, prior to disconnection of the test gas supply until the tracer gas (SF_6) had ‘washed out’ of the lungs. The LCI was calculated by measuring the number of lung turnovers (Cumulative Expired Volume/Functional Residual Capacity) required to attain an end tidal concentration of test gas at 1/40th of the initial ‘washed in’ concentration. Three tests were performed at each visit, with the mean of two or more tests

meeting acceptability criteria used in the final analysis. 14% of the traces were analysed in duplicate by investigators at two sites to ensure consistency; the 95% limits of agreement were -0·04 to 0·04 LCI units.

c) Gas transfer

Transfer factor of the lung for carbon monoxide was measured using a single breath technique (London site: Jaeger Masterscreen, CareFusion, Germany; Edinburgh site: CPL, nSpire Health, England). Subjects, who were seated and wearing a nose clip, were asked to exhale to residual volume, then take a maximal inspiration (required to be >90% vital capacity) of the test gas (medical air with 0·28% carbon monoxide and 9% helium) prior to a breath hold of 10 secs duration and a smooth exhalation. This procedure was repeated at least twice on each test day (minimal interval between tests 4 mins). Duplicate estimates of TLCO were required to be within 10% or 1·0 $mmol.min^{-1}.kPa^{-1}$ (whichever was greater) to meet requirements for inclusion. At least 2 technically acceptable tests from up to 10 attempts were obtained and the mean values used for analysis. The following values were recorded for each test: Single Breath TL_{CO}, Alveolar Volume (VA), Transfer Coefficient (K_{CO}, derived from TL_{CO}/AV) and Inspiratory Vital Capacity (IVC). TL_{CO} and K_{CO} were corrected for most recent haemoglobin (usually obtained on the same day) and expressed as TL_{COc} and K_{COc} using standard formulae.

11. Computed tomography lung scans

High Resolution CT scans were obtained on a 64-channel multidetector scanner (Sensation 16 or 64; Siemens Medical Solutions, Erlangen, Germany). On two occasions (pre dosing and at 28±5 days after dose 12) patients underwent a CT comprising contiguous thin sections (1·25 mm) through the entire volume of the lungs obtained during breath-holding at full inspiration and also interspaced (1 mm sections at 10 mm increments) at end-expiration. A high-spatial-resolution algorithm was used for image reconstruction. The CTs were scored independently by two radiologists with an interest in in thoracic imaging; scans had been anonymized and scores were assigned with the radiologists blinded both to subject identification and whether the CT was obtained pre or post-dosing. All scoring was performed directly from workstations with image manipulation allowed for window settings (default: width 1,500 Hounsfield units, centre -500 Hounsfield units). The presence and severity of specific CT features was recorded for each lobe as previously described.[10] The features studied were extent of bronchiectasis (scored per lobe on a range of 0-3 and the results meaned), severity of bronchiectasis and bronchial wall thickness (scored per lobe on a range of 0-4 and the results meaned), small and large airway mucus plugs (scored per lobe on a range of 0-2 and the results meaned), and gas trapping on expiratory CT (scored on a percentage basis). In addition, as a safety outcome, all subjects underwent a limited cut inspiratory CT scan pre-dosing at the dose 4 visit; these scans were not formally scored, but were reviewed in real time by the clinical radiology team for any acute changes, and reports provided to the DMEC.

12. VO_2 max

Tests were performed on a stationary exercise bike with breath-by-breath analysis. Subjects pedalled at 70 revolutions per minute (rpm) whilst wearing a nose clip and a mouth piece attached to an Innocor gas analyser (Innovision, Odense, DK). Resistance increased each minute from a starting workload which, in children, was dependent on the patient's height and calculated using the Godfrey protocol:

- Patients < 120 cm use 10W starting resistance and 10W increments.
- Patients 120-150 cm use 15W starting resistance and 15W increments.
- Patients > 150 cm use 20W starting resistance and 25W increments.

The subjects' heart rate, oxygen saturations and Borg scale was measured at rest and each minute during exercise whilst patients exercised to their maximum capacity. The Innocor system was validated against standard calibrated VO_2 equipment using paired exercise tests in 12 CF patients. For VO_2 max, the mean difference was -0·026 l/min and the 95% confidence interval was -0·27 to 0·22 l/min (unpublished observations).

13. Activity monitoring

Patients wore the SenseWear^{R} body monitoring system^{TM} (BodyMedia, Pittsburgh, USA) for at least 7 whole days at three time points throughout the trial (pre dosing, dose 6 and post dosing). The device uses accelerometry, heat flux, skin temperature and galvanic skin response sensors to gather physiological data on movement and daily physical activity patterns. These armbands have been validated in normal subjects and a number of patient groups.[11] Data from activity monitors were downloaded and analysed using proprietary software. Final data were analysed when there was at least four days of data, at least one day was a weekend and at least 10 hours of data for each day.

This analysis yields data for the proportion of time that wearers spend at various levels of energy expenditure. For the purposes of summarising these data in the present trial, a comparison was made (between Active and Placebo groups) of the percentage of time spent in vigorous exercise, defined as >3 Metabolic Equivalents (METS), compared to the percentage of time spent at ≤3 METS of activity, as well as the mean number of steps taken per day.

14. Bronchoscopic procedures

All bronchoscopies were performed under general anaesthesia with patients endotracheally intubated, as previously described, by a single operator.[12] Ringers and zero chloride solutions were manufactured by the Pharmacy at Eastbourne Hospital, UK and comprised the chemicals in the Cystic Fibrosis Foundation Therapeutic Development Network (CFF TDN) SOP,[13] with the exception that amiloride was omitted from the latter. They were warmed prior to use to reduce the formation of microbubbles, but were used at room temperature and perfused at 100 ml/hour. Basal (non-stimulated) PD was measured on each wall of the distal trachea with sterile Ringer's solution. Where possible, a stable period (<1 mV change over 30 seconds) was recorded. Subsequently, at three sites more distally (approximately 5th generation airways), following a stable 30 second period with Ringer's solution, the solution was switched to a zero-chloride equivalent containing isoprenaline (10 µM) for 5 min. The catheter was removed and re-primed between measurements. Hardware and software were as previously described.[12] Ten bronchial brushing samples were obtained from throughout the airways of the same lung using disposable cytology brushes (BC-202D-5010, Olympus UK, Southend-on-Sea, UK). They were placed into PBS and processed for DNA/ mRNA analysis (see below). Two endobronchial biopsies were obtained from approximately 5th generation subcarinae with disposable forceps (BC-202D-5010, Olympus UK, Southend-on-Sea, UK) and processed as described below. Bronchoalveolar lavage was not undertaken, but where possible, samples of secretions were sent for microbiological culture. Patients were observed for 4-6 hr prior to discharge. One bronchoscopy was undertaken prior to dosing and one 28±5 days after the 12th dose. Four patients had measurements taken beyond these time windows due to clinical instability: 3 infective exacerbations (1 Active, 2 Placebo) and one acute pancreatitis (Active). In addition, one subject who wished to withdraw from the trial agreed to have his bronchoscopy after his dose 6, but within the defined window.

Bronchial potential recordings (1-3) from the same patient at the same time point were pre-grouped and scored blinded both for acceptability and response. Recordings were only accepted for scoring if a) the time of any inflexion due to the zero chloride/isoproterenol solution was approximately 50-80 seconds after onset of perfusion, this duration having previously been shown to represent clearing of the dead space within the catheter (~50 secs) and allowing ~30 secs for the initiation of any isoprenaline response and b) the response fell into one of three categories namely no response, continuous upward (hyperpolarisation), or continuous downward (depolarisation) deflection. Recordings were excluded if a) the response at the 50-80 sec time point was unstable (rising or falling), or b) the deflection was characterised by both a depolarisation and a hyperpolarisation. Traces were scored by four investigators with experience in the technique (EWFWA, JCD, KH and SNS). Overall 66/102 (64·7%) recordings fulfilled the acceptability criteria.

15. Nasal brushing

Nasal brushings were performed in subjects in the nasal subgroup both pre-dosing and at 28+/-5 days post final dose, from under the middle or inferior turbinate of both nostrils using interdental brushes (Dent-o-care, London, UK). Cells were suspended in 800 µl of PBS and processed for transgene DNA/mRNA analysis (see below).

16. Quantification of DNA and mRNA

DNA and RNA were simultaneously isolated from nasal and bronchial brushing samples using AllPrep (QIAGEN, Manchester, UK). Levels of pGM169-specific and endogenous *CFTR* DNA and mRNA were quantified using TaqMan (Life Technologies, Paisley, UK) real-time quantitative PCR instruments and supplies, following reverse transcription (RT) where appropriate, essentially as described by Rose *et al* 2002.[14] Absolute DNA and *in vitro* transcribed RNA standards allowed precise copy number quantification. pGM169 DNA levels were determined using a qPCR primer set specific for the soCFTR2 cDNA:
50 nM forward primer: 5'-GGAACAGCTCCAAGTGCAAGA-3'
900 nM reverse primer: 5'-CCTGGTGTCCTGCACTTCCT-3'
100 nM probe: 5'-FAM-CAAGCCCCAGATTGCTGCCCTG-TAMRA-3'.

Levels were normalised to total genomic DNA as determined using a qPCR primer set specific for the endogenous CFTR gene:
300 nM forward primer 5'- CTTCCCCCATCTTGGTTGTTC-3'
300 nM reverse primer: 5'-TGACAGTTGACAATGAAGATAAAGATGA-3'
100 nM probe: 5'-VIC-TGTCCCCATTCCAGCCATTTGTATCCT-TAMRA-3'.

pGM169-derived mRNA was determined using qRT-PCR. Reverse transcription reactions (20 μl) contained ≤5 μl (≤500ng) total RNA, 1·25 units of MultiScribe Reverse Transcriptase (LifeTechnologies) and 0·4 Units RNAse inhibitor (LifeTechnologies) and 400nM of the appropriate primer:
soCFTR2 mRNA: 5'-CCAGCTGAAGAACAGCTTGCT-3'
hCFTR mRNA: 5'-CCAGCTGAAGAACAGCTTGCT-3'.

pGM169-derived cDNA levels were determined using a qPCR primer set specific for the correctly spliced exon 1-2 soCFTR2 mRNA:
300 nM forward primer: 5'-TCTCCCTCCTGTGAGTTTGGTT-3'
300 nM reverse primer: 5'-GCTCACCACAGAGGCCTTCT-3'
100 nM probe: 5'-FAM-CTAGCCACCATGCAGAGAAGCCCTCTG-TAMRA-3'.

Levels were normalised to endogenous hCFTR mRNA as determined using a qPCR primer set specific for the correctly spliced exon 1-2 endogenous *CFTR* mRNA:
300 nM forward primer: 5'-GGAAAAGGCCAGCGTTGTC-3'
300 nM reverse primer: 5'-CCAGGCGCTGTCTGTATCCT-3'
100 nM probe: 5'-VIC-CCAAACTTTTTTTCAGCTGGACCAGACCAA-TAMRA-3'.

qPCR reactions (10 μl) contained 0·6-6 ng total DNA or 2 μl cDNA and 1xTaqMan Universal MasterMix (LifeTechnologies). Thermocycling was performed in 384-well plates, on a 7900HT cycler, with SDS 2·2 software, and the following cycling conditions: 50°C 2 min, 95°C 10 min, then 40 cycles of 95°C 15 sec, 60°C 1 min. Data were calculated as the percentage of pGM169-specific to endogenous CFTR copy number. Where samples were positive for pGM169-specific signal, but either quantification fell below the linear range of the standards (LOQ - Limit Of Quantification) or quantification of endogenous CFTR signal was negative, they were scored as Positive But Not Quantifiable (PBNQ). Remaining samples that were negative for endogenous CFTR signal were scored as Not Determined (ND). Samples negative for pGM169-specific signal but positive for endogenous CFTR signal were scored as zero. Where data are presented as post-pre treatment difference, a conservative approach was adopted for samples scored with the nominal value of PBNQ by substituting a value of either 0 or the LOQ as appropriate to maximise the post-pre difference for Placebo samples and minimise the post-pre difference for Active samples. For bronchial brushings, two nominally identical samples were typically available (each pools of five independent bronchial brushings) and the maximum score was reported.

17. Nasal potential difference

At the time of the first measurement in any subject, a single use, disposable, dual-lumen catheter (Marquat, France) was fixed in place at the site of maximal unstimulated PD in one nostril; for all subsequent measurements, the same nostril and the same distance into the nasal cavity was used. Solutions comprised chemicals listed in the CF TDN SOP,[13] and were perfused at room temperature a rate of 4 ml/min in the following sequence: Ringers, Ringers containing amiloride (0·1 mM), zero chloride Ringers containing amiloride and finally zero chloride Ringers containing amiloride and isoprenaline (10 μM). Where possible, three traces were obtained on different days pre-dosing. Two measurements were made post-dosing, one after

14±2 days and one after 28±5 days. Five patients (4 Active, 1 Placebo) had measurements taken beyond these time windows for clinical reasons (maximum of 29 days for FU1 and 72 days for FU2).

Traces were scored by 4 investigators experience in the technique (EWFWA, JCD, SNS and MDW). The 4 components (baseline, amiloride, zero chloride and isoprenaline responses) were scored individually using standard criteria.

18. Statistical Analysis Plan

A detailed statistical analysis plan (SAP) was drafted by the trial statistician (GDM) and was further refined with input from the Trial Management Group. The SAP was approved by the Trial Steering Committee and finalised ahead of the database being locked and the trial unblinded. Data management was undertaken using Excel (Microsoft Version 14·4·6 for Mac OSX). Descriptive statistics and standard analyses were performed using Prism (Version 5·0c for Mac OSX) and/or IBM SPSS Statistics (Version 22·0). There was no exploration of the impact of missing values in the primary analysis since data were available for 114 out of the 116 PP patients. A copy of the SAP has been lodged with the Journal, and is available on request from the corresponding author.

RESULTS

1. Reasons for discontinuation, once subjects were randomized, prior to dose 1

Four subjects were discontinued prior to receiving their first dose of trial medication. Three (2 Active, 1 Placebo) reconsidered participation/withdrew consent and 1 (Placebo) was clinically unstable.

2. Reasons for discontinuation once subjects were enrolled in the ITT population

Placebo (n=6)

Time commitments (2), Venepuncture dislike (1), Increased respiratory adverse events (1), New culture of *Mycobacterium abscessus* (1) and commenced ivacaftor (1).

Active (n=14)

Time commitments (5), Wish to discontinue contraception (1), Changed mind regarding participation (1), Borderline FEV_1 at Screening and throughout study (1), Lived in nomadic community and not available for follow up (1), New culture of MRSA (1), New culture of *Mycobacterium abscessus* (1), New culture of *Burkholderia cepacia*(1) and commenced ivacaftor (2).

3. Interim safety analysis

On two occasions during the trial a) when 17 subjects had received three doses each and b) when all patients had received ≥ five doses, the independent DMEC reviewed all safety data in an unblinded fashion including post-Dose 4 limited-cut CT scans, and confirmed that the trial could continue. There were no interim analyses for efficacy.

4. ITT intra-group analysis

For the ITT groups the relative changes in FEV_1 within each treatment group were: Placebo -3·9% (CI -6·3 to -1·4) and Active -0·3% (CI -2·6 to 2·0).

5. Sputum microbiology

There were no clinically relevant changes in sputum microbiology including: *Aspergillus fumigatus, Haemophilus influenzae*, *Mycobacteria*, *Pseudomonas aeruginosa* and *Staphylococcus aureus*.

6. Subgroup analysis

We assessed whether secondary outcome measures also suggested a stratified response with respect to more or less severe baseline FEV_1 at trial entry (Fig S5). A similar effect to that on the primary outcome was seen in the majority of the assays. The more severe subgroup showed an approximate doubling of the standardised treatment effect compared with the respective values in the unstratified group. This resulted in an absolute improvement (versus stabilization) in some of the assays. Of note, in those with less severe FEV_1 at trial entry, biomarkers associated with smaller airways, in particular Lung Clearance Index (LCI), but also FVC, CT small airway mucus plugging and gas trapping still showed a treatment effect favouring the Active group (Fig S5). Antibiotic values for the more severe half, stratified by FEV_1, were: Placebo 3, Active 3 and for the less severe half: Placebo 3 Active 2.

7. CFTR molecular outcomes in nasal arm of sub-study

One patient had a total chloride secretory response ≥ 5 mV pre-dosing and was, therefore, excluded as pre-specified in the Protocol. Day 28 post-treatment recordings for two Active patients had to be delayed beyond a window of >7 days of the pre-specified interval and were therefore excluded; both patients had usable values at the Day 14 time point post-treatment. Overall, 75/106 (70·8%) of zero chloride, and 70/106 (66·0%) of isoprenaline recordings were interpretable. There were no significant changes in baseline values or amiloride responses in either the Placebo or Active groups. Placebo (n=6): Baseline mean (SEM) predosing -51·4 mV (7·5), postdosing -45·8 mV (7·6), amiloride response predosing +31·1 mV (7·9), postdosing +29·5 mV (6·9). Active (n=14): Baseline mean (SEM) predosing -54·5 mV (3·9), postdosing -47·0 mV (4·1), amiloride response predosing +31·3 mV (3·1), postdosing +26·0 mV (3·2).

For the zero chloride component 10 of 14 Active and 3 of 6 Placebo patients showed net secretion (ie a negative value); 4 of 14 Active patients showed mean pre-post treatment responses (ranging from -3·4 to -7·0 mV), which were more negative than the largest Placebo response (Fig S8b). For the isoprenaline component, 11 of 12 Active and 4 of 6 Placebo patients showed net secretion (Fig S8c).

8. CFTR molecular outcomes in bronchial arm of sub-study

In the bronchial subgroup, one Active patient withdrew after six doses but consented to post-dosing bronchoscopy within the pre-specified timing (28 ± 5 days) after the last dose; values for this patient are included in the analysis. Two Placebo patients had their post-bronchoscopy 58 and 62 days, and two Active patients 49 and 111 days after their last dose. The data for the two Placebo patients are included in the final analysis on the basis that the natural history of bronchial electrophysiology is unlikely to be influenced by a dose of saline approximately 60 days previously. Values for the Active two patients were omitted from the final analysis given the monthly dosing schedule in the trial based on previously generated gene expression data. There were no significant changes in baseline values (mean of tracheal and all bronchial measurements for any subject) in either the Placebo group: Predosing-17·8 mV (SEM 2·1), postdosing -15·7 mV (1·3) or Active group: Predosing -15·8 mV (1·7), postdosing -14·0 mV (1·7).

9. Adverse events

Previous Phase I/IIa plasmid DNA liposome gene therapy studies have highlighted the potential for flu-like symptoms within the first 48 hours following dosing. Here, one patient in the Placebo group and one in the Active noted these symptoms, predefined as occurring following ≥4 treatment doses. Similarly, we have previously noted a restrictive drop in spirometry in the first few hours following nebulisation, unresponsive to β_2-agonists and self-limiting. In this trial, spirometry was not performed post-dosing to limit the potential for unblinding. However, lower airway symptoms were again noted following ≥4 doses in 11·3% of Placebo patients (7 of 54) and in 25·8% of Active patients (16 of 62, p=0·104, Fisher's Exact Test).

No clinically relevant changes were noted in weight, BMI, serum haematology and biochemistry, urine analysis, sputum/cough swab/bronchial aspirate microbiology, or sputum inflammatory markers.

REFERENCES

1. Eastman SJ, Tousignant JD, Lukason MJ, et al. Optimization of formulations and conditions for the aerosol delivery of functional cationic lipid:DNA complexes. *Hum Gene Ther* 1997; **8**(3): 313-22.
2. Hyde SC, Pringle IA, Abdullah S, et al. CpG-free plasmids confer reduced inflammation and sustained pulmonary gene expression. *Nat Biotechnol* 2008; **26**(5): 549-51.
3. Davies LA, Nunez-Alonso GA, Hebel HL, et al. A novel mixing device for the reproducible generation of nonviral gene therapy formulations. *Biotechniques* 2010; **49**(3): 666-8.
4. Davies LA, Nunez-Alonso GA, McLachlan G, Hyde SC, Gill DR. Aerosol delivery of DNA/liposomes to the lung for cystic fibrosis gene therapy. *Hum Gene Ther Clin Dev* 2014; **25**(2): 97-107.
5. Derand R, Bulteau-Pignoux L, Becq F. Comparative pharmacology of the activity of wild-type and G551D mutated CFTR chloride channel: effect of the benzimidazolone derivative NS004. *J Membr Biol* 2003; **194**(2): 109-17.
6. Horsley AR, Davies JC, Gray RD, et al. Changes in physiological, functional and structural markers of cystic fibrosis lung disease with treatment of a pulmonary exacerbation. *Thorax* 2013; **68**(6): 532-9.
7. Lian X, Yan C, Yang L, Xu Y, Du H. Lysosomal acid lipase deficiency causes respiratory inflammation and destruction in the lung. *Am J Physiol Lung Cell Mol Physiol* 2004; **286**(4): L801-7.
8. Calcedo R, Griesenbach U, Dorgan DJ, et al. Self-reactive CFTR T cells in humans: implications for gene therapy. *Hum Gene Ther Clin Dev* 2013; **24**(3): 108-15.
9. Stanojevic S, Wade A, Stocks J. Reference values for lung function: past, present and future. *Eur Respir J* 2010; **36**(1): 12-9.
10. Irving SJ, Ives A, Davies G, et al. Lung clearance index and high-resolution computed tomography scores in primary ciliary dyskinesia. *Am J Respir Crit Care Med* 2013; **188**(5): 545-9.
11. Johannsen DL, Calabro MA, Stewart J, Franke W, Rood JC, Welk GJ. Accuracy of armband monitors for measuring daily energy expenditure in healthy adults. *Med Sci Sports Exerc* 2010; **42**(11): 2134-40.
12. Davies JC, Davies M, McShane D, et al. Potential difference measurements in the lower airway of children with and without cystic fibrosis. *Am J Respir Crit Care Med* 2005; **171**(9): 1015-9.
13. Rowe SM, Clancy JP, Wilschanski M. Nasal potential difference measurements to assess CFTR ion channel activity. *Methods Mol Biol* 2011; **741**: 69-86.
14. Rose AC, Goddard CA, Colledge WH, Cheng SH, Gill DR, Hyde SC. Optimisation of real-time quantitative RT-PCR for the evaluation of non-viral mediated gene transfer to the airways. *Gene Ther* 2002; **9**(19): 1312-20.

SUPPLEMENTARY FIGURE AND TABLE LEGENDS

Figure S1

Time course of the response of FVC to either Placebo (red circles) or Active (blue squares). 'Pre' and 'Post' indicate the mean of two measurements at the respective time points. Error bars indicate SEM. There was a significant (p=0·031) treatment effect, with the Active group having an ANCOVA-adjusted improvement of 3·0% (95% CI 0·3-5·8) compared to Placebo at 12 months follow-up.

Figure S2

Forest plot showing stratification of the primary outcome response by pre-specified variables. To allow results from different endpoints to be plotted on a common scale, the estimated treatment effects were standardised to be presented as multiples of the underlying standard deviation (Standardised Treatment Effect). The size of the circle is proportional to the number of subjects represented and the bars indicate 95% CI. Assay sample numbers, pre-treatment values, the treatment effect (post-pre change) in absolute assay values and ANCOVA interaction p values are also shown for reference. For all CT values and LCI, lower numbers indicate less severe disease. Con Meds=Concurrent Medication. MP=Mucus Plugs. Pa=*Pseudomonas aeruginosa*. TA AE=Treatment Associated Adverse Event. Note the values for FEV_1 shown are the relative change in % predicted FEV_1.

Figure S3

Forest plot showing the responses of the Placebo (red) or Active (blue) arms when assessed by the pre-defined sub-group values shown. To allow results from different endpoints to be plotted on a common scale, the estimated treatment effects were standardised to be presented as multiples of the underlying standard deviation. The size of the circle is proportional to the number of subjects represented and the bars indicate 95% CI. Assay sample numbers are shown for reference. The interaction p values from the ANCOVA model are shown for all analyses except for the primary endpoint, which is shown in square brackets to highlight this difference. Con Meds=Concurrent Medication. MP=Mucus Plugs. Pa=*Pseudomonas aeruginosa*. TA AE=Treatment Associated Adverse Event.

Figure S4

Time course of the primary outcome response stratified by baseline FEV_1 at trial entry. Red circles indicate the Placebo group and blue squares the Active group. Error bars indicate SEM:

A. Baseline FEV_1 49·6-69·2% predicted
B. Baseline FEV_1 69·6-89·9% predicted

Figure S5

Forest plot showing stratification of secondary outcomes by the severity of baseline FEV_1 at trial entry. To allow results from different endpoints to be plotted on a common scale, the estimated treatment effects were standardised to be presented as multiples of the underlying standard deviation (Standardised Treatment Effect). The size of the circle is proportional to the number of subjects represented and the bars indicate 95% CI. Assay sample numbers are shown for reference. The interaction p values from the ANCOVA model are shown for all analyses except for the primary endpoint, which is shown in square brackets to highlight this difference. QoL Physical & QoL Respiratory = Health Related Quality of Life CFQ-R Physical Functioning and Respiratory Symptoms respectively.

Figure S6

Forest plot showing the responses of exploratory outcome measures to treatment. To allow results from different endpoints to be plotted on a common scale, the estimated treatment effects were standardised to be presented as multiples of the underlying standard deviation (Standardised Treatment Effect), with positive values indicating an improvement towards non-CF values. Assay sample numbers, pre-treatment values, the treatment effect (post-pre change) in absolute assay values and ANCOVA adjusted p value differences are also shown for reference. The size of the circle is proportional to the number of subjects represented and the bars indicate 95% CI.

Figure S7

Absolute values of bronchial potential difference in subjects with both pre- (prior to first dose) and post- (nominally 28 days after twelfth dose) samples. Dots represent individual values, lines connect pre- and post- pairs.

A. Mean absolute change in bronchial potential difference in response to combined zero chloride and isoprenaline (10 µM) in Placebo subjects.
B. Mean absolute change in bronchial potential difference in response to combined zero chloride and isoprenaline (10 µM) in Active subjects.
C. Maximum (most negative) absolute change in bronchial potential difference in response to combined zero chloride and isoprenaline (10 µM) in Placebo subjects.
D. Maximum (most negative) absolute change in bronchial potential difference in response to combined zero chloride and isoprenaline (10 µM) in Active subjects.

Figure S8

A. Assessment of DNA from nasal brushings in the Placebo (n=6) and Active (n=17) sub-groups. Each circle represents an individual patient. LOQ=Limit of quantification, PBNQ=Positive but not quantifiable. Bar = median.
B. The response of the nasal epithelium to perfusion with a zero chloride solution. Each symbol indicates the change in this response from trial start to finish for the relevant treatment in an individual patient. The plotted value is the mean of all interpretable recordings (range 1-3) at each time point for that patient. A more negative value is in the non-CF direction. The Placebo group had a median pre-post trial change of +0·1 mV, range +1·1 to -2·3 mV and the Active group -0·6 mV, range +3·5 to -7·0, p=0·509.
C. The response of the nasal epithelium to perfusion with a zero chloride solution containing isoprenaline (10 µM). Each symbol indicates the change in this response from trial start to finish for the relevant treatment in an individual patient. The plotted value is the most negative value obtained from all interpretable recordings (range 1-3) at each time point for that patient. A more negative value is in the non-CF direction. The Placebo group showed a median pre-post trial change of +0·3 mV, range +2·9 to -2·6 mV and the Active group -1·0 mV, range +0·1 to -3·8 mV, p=0·424.

Figure S9

Absolute values of nasal potential difference in subjects with both pre- (prior to first dose) and post-(nominally 28 days after twelfth dose) samples. Dots represent individual values, lines connect pre- and post- pairs.

A. Mean absolute change in nasal potential difference in response to zero chloride in Placebo subjects.
B. Mean absolute change in nasal potential difference in response to zero chloride in Active subjects.
C. Mean absolute change in nasal potential difference in response to isoprenaline (10 µM) in Placebo subjects.
D. Mean absolute change in nasal potential difference in response to isoprenaline (10 µM) in Active subjects.

Figure S10

Histological assessment of bronchial biopsies. Bronchial biopsies were collected before the first dose (Pre) and 28±5 days after the last dose (Post). Sections were stained with haematoxylin and eosin. 39 of the 50 possible biopsies (78%) were analysed. The remaining 11 were not analysable because of poor sample quality (n=10) or because the patient withdrew from the trial (n=1). For the final analysis only patients with available pre- and post-treatment biopsies were analysed, and the reported numbers for each parameter vary due to sample quality (for example goblet cell hyperplasia was not quantifiable in all samples due to sample denudation). Sections were scored semi-quantitatively using a scale from 0-6, except for seromucinous gland hyperplasia which was scored as absent (0)/present (1). All data are expressed as the change from post minus pre-treatment. A positive number indicates an increased score following treatment. Dots represent scores for individual biopsies. The horizontal bar indicates the median.

A: Goblet cell hyperplasia
B: Basement membrane thickening
C: Numbers of lymphocytes
D: Numbers of neutrophils
E: Numbers of eosinophils
F: Seromucinous gland hyperplasia

Table S1

Details of the six Serious Adverse Events in the Active group.

Table S2

Quantification of CFTR-specific T-cells in the Per Protocol (PP) population.

Peripheral blood mononuclear cells (PBMCs) were extracted before the first dose (Pre-dose) and 2 or 4 weeks after the last dose (Follow-up) and a human interferon-γ enzyme-linked immunospot assay was carried out. Samples were classified as positive or negative for the presence of CFTR-specific T cells. Where recovery of viable PBMC was too low to perform analysis, samples were classified as “Not determined”. *indicates a Placebo subject negative for CFTR-specific T cells before dosing and T-cell converted to positive after the last dose. ** indicates an Active subject positive for CFTR-specific T-cells before dosing and T-cell converted to negative after the last dose.

Table S3

Quantification of anti-dsDNA antibodies

Anti-double-stranded (ds) DNA antibodies were quantified in serum before the first dose (Pre) and 4 weeks after the last dose (Post). Samples were classified as positive or negative. When blood samples could not be obtained, these are indicated as “Not determined”.

Table S4: Quantification of lipid-laden cells in bronchial biopsies and sputum

A. Bronchial biopsies were collected before the first dose (Pre) and 28±5 days after the last dose (Post). Lipid-laden macrophages were quantified in 38 of the 50 possible biopsies (76%). The remaining 12 biopsies were not analysable because of poor sample quality (n=7) or because the patients withdrew from the trial (n=5). Tissue sections were scored semi-quantitatively as containing 0, 1-6, 7-14 or ≥15 lipid-laden macrophages/section
B. Differential counts of lipid-staining cells present in sputum samples from Placebo and Active treated subjects. ANCOVA-adjusted mean values, together with the 95% Confidence Interval in square brackets, are shown. Numbers in round brackets indicate analysable samples.

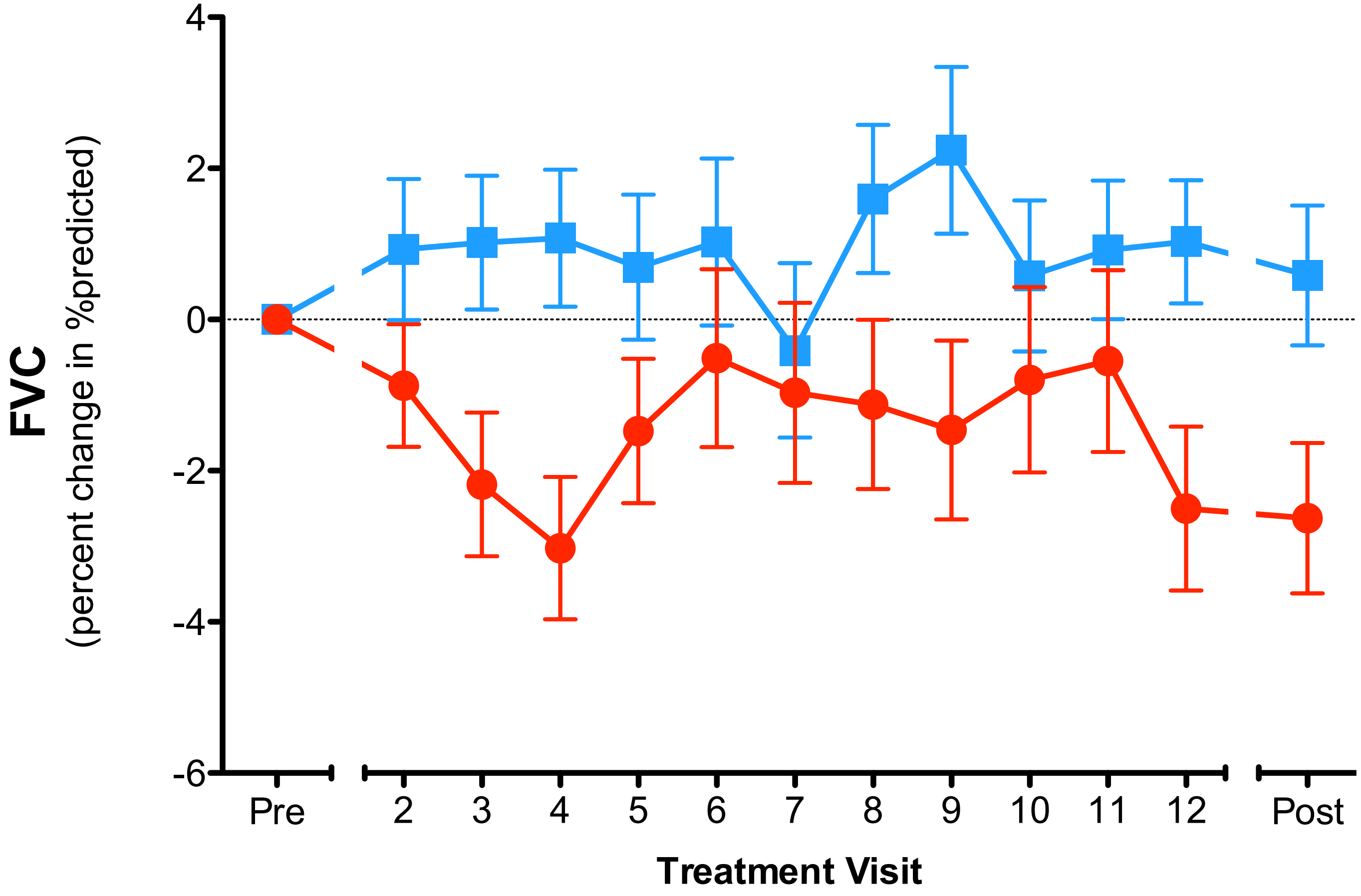

Alton *et al* Figure S1

		N Placebo/Active	Pre-Treatment Mean (SD) [Median]	Absolute Treatment Effect Mean [95% CI]	Standardised Treatment Effect Mean [95% CI]	Interaction P value
**Primary Endpoint**	All	54/60	69·48 (10·51) [69·42]	3·66 [0·07 to 7·25]	0·39 [0·01 to 0·77]	[0·046 *]
**FEV_1**	49.6 - 69.2 % predicted	29/27	60·58 (5·27) [60·66]	6·42 [0·76 to 12·08]	0·69 [0·09 to 1·29]	0·065
	69.6 - 89.9 %predicted	25/33	78·38 (5·81) [77·89]	0·19 [-4·56 to 4·93]	0·02 [-0·49 to 0·53]	
**LCI**	6.97 - 10.47 LCI	23/34	74·36 (8·69) [75·18]	5·26 [-0·10 to 10·61]	0·56 [-0·01 to 1·13]	0·453
	10.55 - 19.64 LCI	29/25	64·33 (9·58) [63·01]	1·87 [-3·65 to 7·38]	0·20 [-0·39 to 0·79]	
**CT**	0.17 - 1.92 Wall Thickness	26/34	72·97 (10·43) [73·97]	3·71 [-0·96 to 8·37]	0·40 [-0·11 to 0·89]	0·787
	2.00 - 3.00 Wall Thickness	28/26	65·98 (9·24) [66·72]	3·68 [-2·24 to 9·59]	0·39 [-0·24 to 1·02]	
	0.00 - 0.92 Large Airway MP	26/32	73·75 (10·26) [74·70]	3·18 [-2·31 to 8·66]	0·34 [-0·25 to 0·92]	0·707
	1.00 - 1.50 Large Airway MP	28/28	65·43 (8·91) [66·37]	4·68 [0·02 to 9·33]	0·50 [0·01 to 0·99]	
	0.00 - 0.92 Small Airway MP	21/34	74·42 (9·97) [75·18]	3·38 [-1·50 to 8·25]	0·36 [-0·16 to 0·88]	0·992
	1.00 - 1.58 Small Airway MP	33/26	65·23 (8·87) [65·16]	3·98 [-1·71 to 9·65]	0·43 [-0·19 to 1·03]	
	7.50 - 46.67 Gas Trapping	22/36	74·19 (9·51) [75·10]	4·47 [-0·76 to 9·69]	0·48 [-0·09 to 1·03]	0·831
	47.08 - 71.25 Gas Trapping	32/24	64·98 (9·29) [64·70]	3·51 [-1·99 to 9·01]	0·38 [-0·22 to 0·96]	
**Age**	<18	17/23	72·38 (9·76) [72·02]	5·73 [-1·97 to 13·42]	0·61 [-0·21 to 1·43]	0·287
	≥18	37/37	67·96 (10·63) [67·58]	2·51 [-1·41 to 6·43]	0·27 [-0·15 to 0·69]	
**Gender**	Female	25/29	69·21 (9·59) [69·42]	3·22 [-2·24 to 8·67]	0·35 [-0·24 to 0·92]	0·252
	Male	29/31	69·74 (11·38) [68·61]	1·80 [-3·09 to 6·67]	0·20 [-0·33 to 0·71]	
**Mutation**	F508del/F508del	26/30	68·28 (10·10) [66·81]	3·05 [-2·97 to 9·07]	0·33 [-0·32 to 0·97]	0·625
	Other	28/30	70·64 (10·85) [71·27]	3·73 [-0·72 to 8·16]	0·40 [-0·08 to 0·87]	
**Pseudomonas**	Pa Free At Entry	27/32	72·68 (9·33) [72·02]	5·83 [1·01 to 10·66]	0·62 [0·11 to 1·13]	0·291
	Pa Colonised At Entry	27/28	66·06 (10·7) [66·36]	1·24 [-4·51 to 6·98]	0·14 [-0·48 to 0·74]	
**Con Meds**	Not Using Azithromycin	16/18	72·15 (10·74) [73·52]	2·94 [-4·95 to 10·81]	0·32 [-0·53 to 1·15]	0·614
	Using Azithromycin	38/42	68·33 (10·26) [66·89]	3·65 [-0·46 to 7·74]	0·39 [-0·05 to 0·83]	
	Not Using Corticosteroids	18/22	71·92 (11·04) [73·52]	5·62 [-0·67 to 11·9]	0·60 [-0·08 to 1·27]	0·980
	Using Corticosteroids	36/38	68·15 (10·03) [66·91]	3·84 [-1·03 to 8·70]	0·41 [-0·11 to 0·93]	
	Not Using DNase	19/24	71·87 (11·32) [73·52]	1·17 [-4·82 to 7·16]	0·13 [-0·52 to 0·76]	0·549
	Using DNase	35/36	68·07 (9·81) [66·89]	4·15 [-0·81 to 9·10]	0·44 [-0·09 to 0·97]	
	Not Using Hypertonic Saline	38/38	70·94 (10·50) [71·66]	3·62 [-0·21 to 7·45]	0·39 [-0·03 to 0·79]	0·778
	Using Hypertonic Saline	16/22	66·61 (10·05) [66·54]	3·19 [-5·22 to 11·6]	0·34 [-0·56 to 1·23]	
**TA AEs**	<4 Lower Airway TA AEs	46/45	69·63 (10·35) [69·64]	3·95 [-0·29 to 8·19]	0·42 [-0·04 to 0·87]	0·649
	≥4 Lower Airway TA AEs	8/15	68·89 (11·34) [68·48]	1·30 [-6·29 to 8·89]	0·14 [-0·67 to 0·95]	

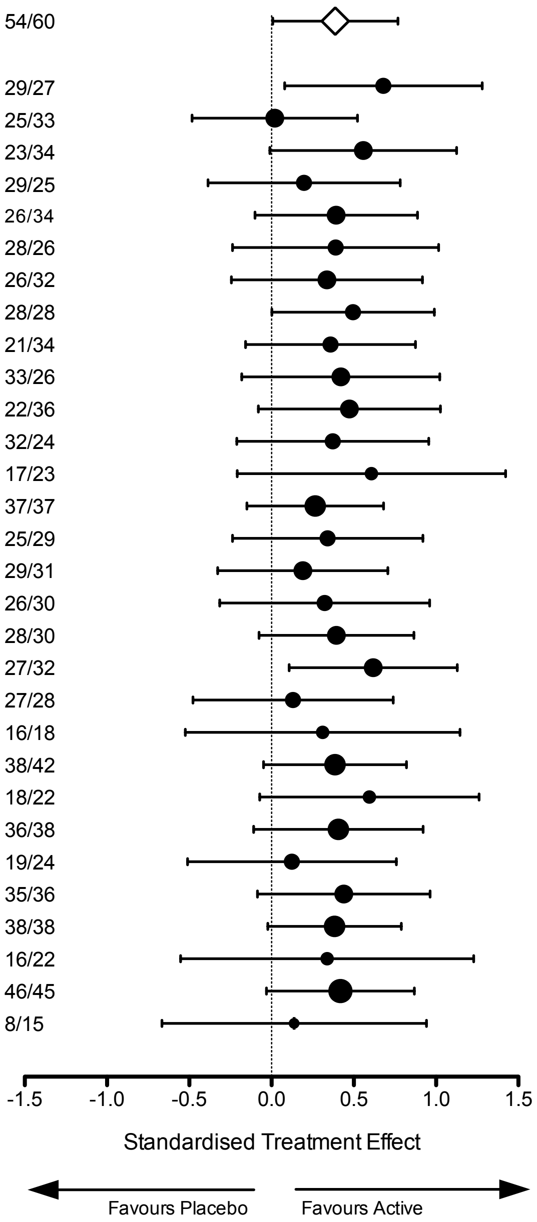

Alton *et al* Figure S2

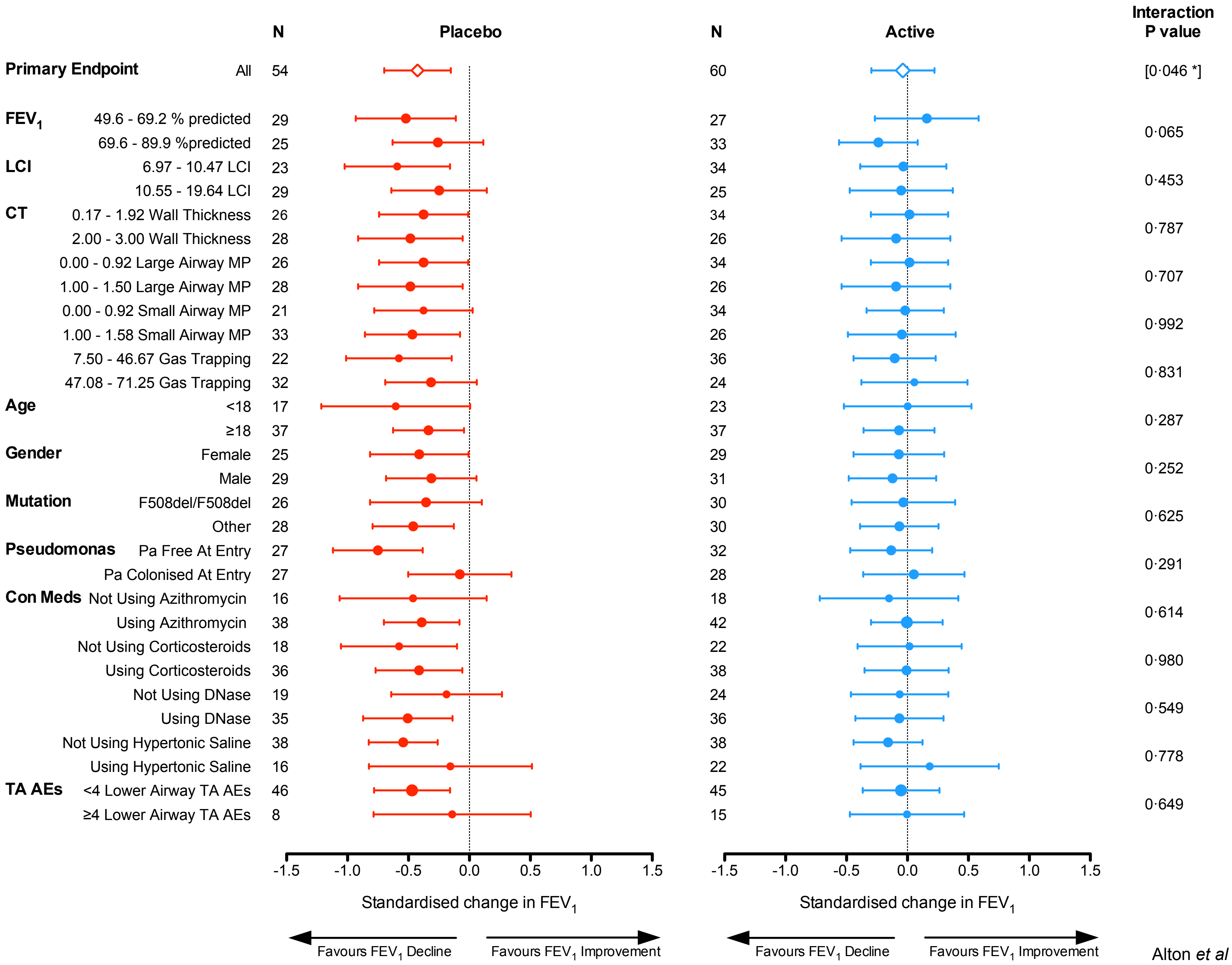

Alton *et al* Figure S3

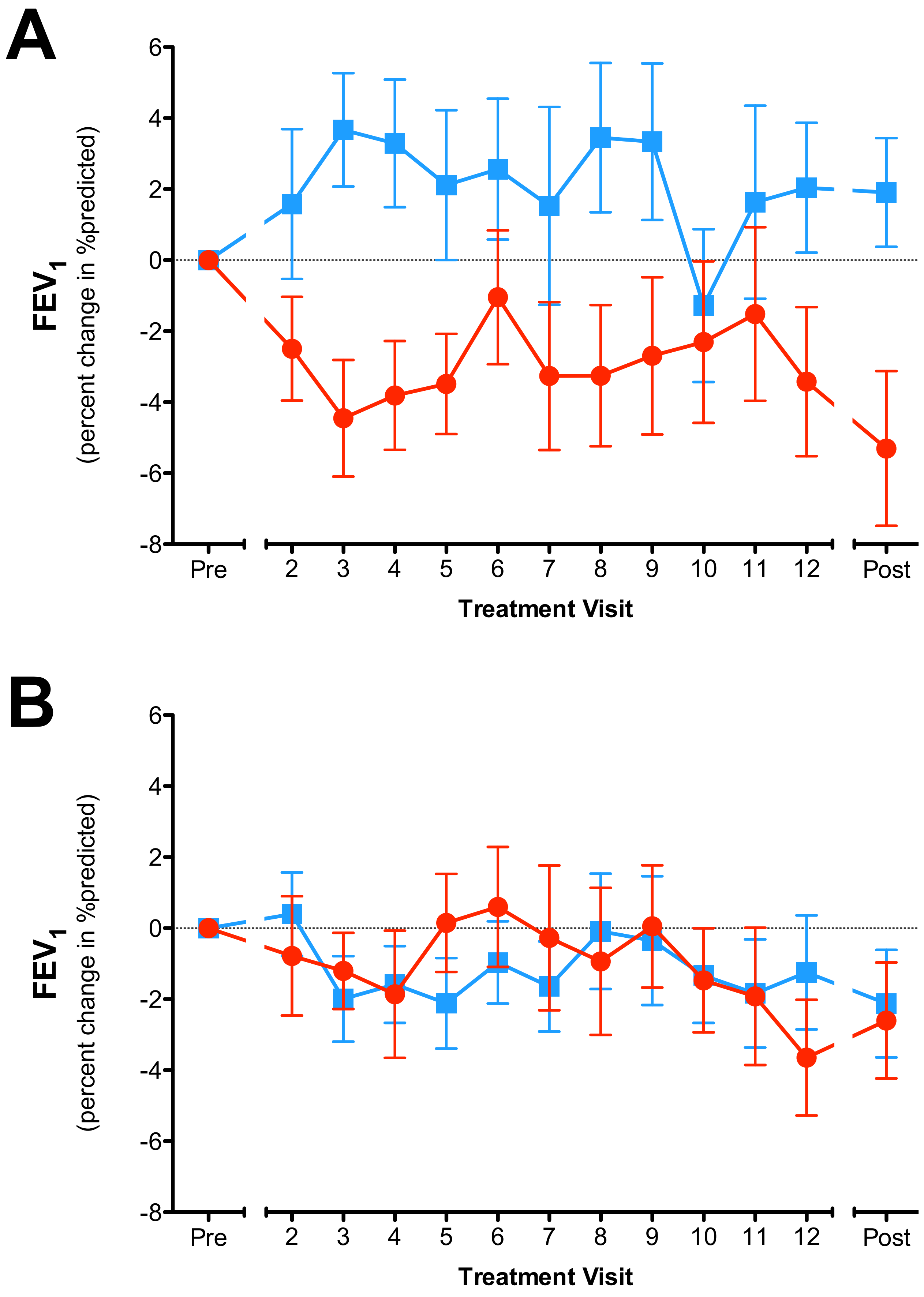
A
6
4
2
0
-2
-4
-6
-8
FEV1
(percent change in %predicted)
Pre
2
3
4
5
6
7
8
9
10
11
12
Post
Treatment Visit
B
6
4
2
0
-2
-4
-6
-8
FEV1
(percent change in %predicted)
Pre
2
3
4
5
6
7
8
9
10
11
12
Post
Treatment Visit

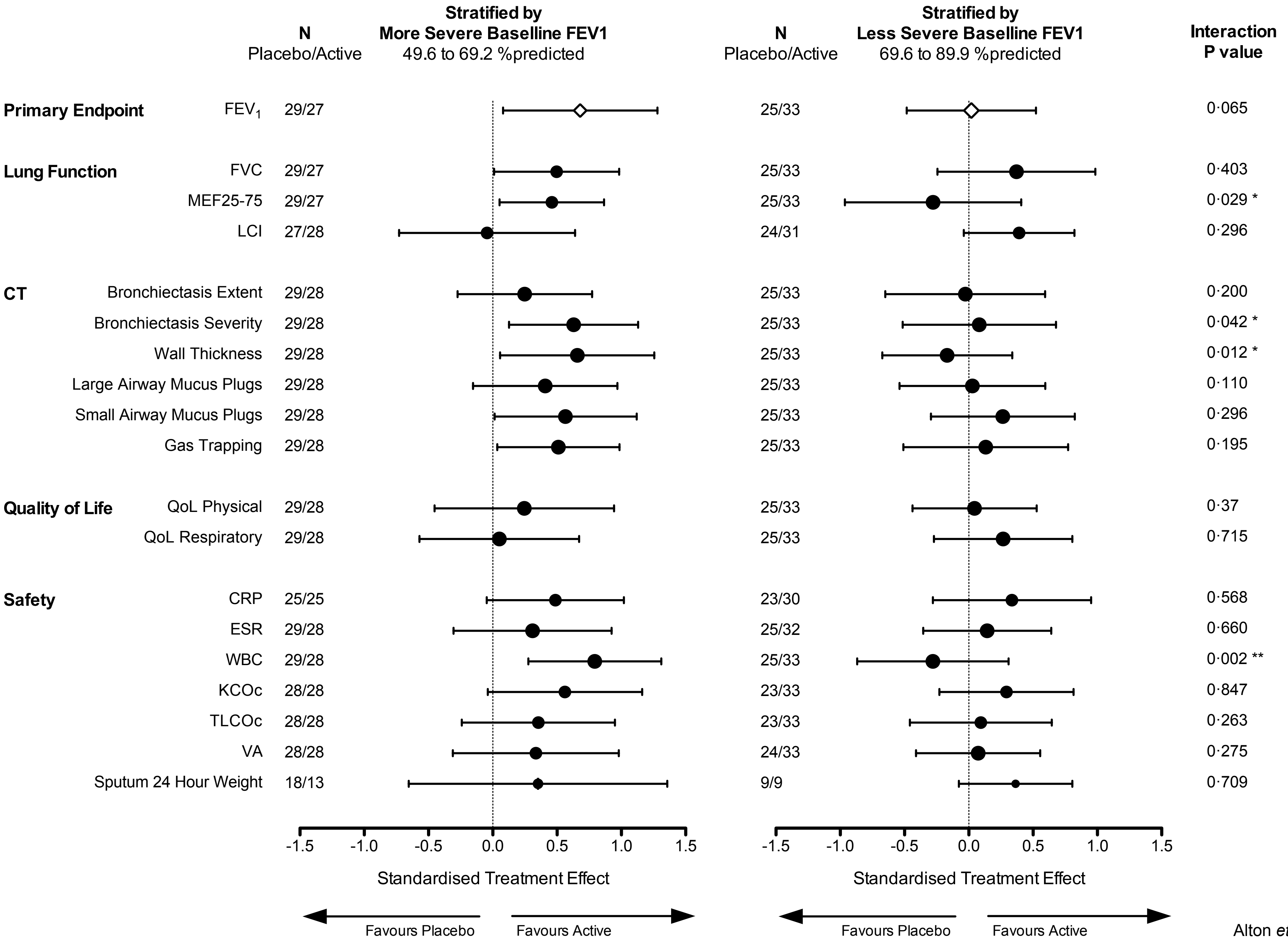

Alton *et al* Figure S5

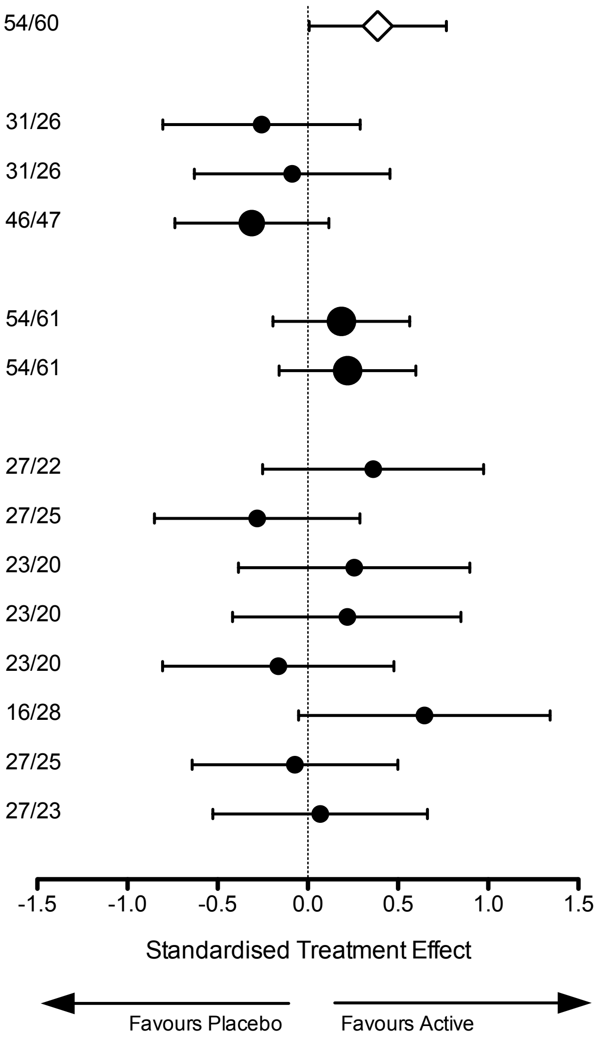

		N Placebo/Active	Pre-Treatment Mean (SD) [Median]	Absolute Treatment Effect Mean [95% CI]	Standardised Treatment Effect Mean [95% CI]	Adjusted P value
**Primary Endpoint**	FEV_1	54/60	69·48 (10·51) [69·42]	3·66 [0·07 to 7·25]	0·39 [0·01 to 0·77]	0·046 *
**Activity & Exercise**	>3 Mets	31/26	155·17 (78·83) [143]	-15·34 [-48·03 to 17·36]	-0·26 [-0·81 to 0·3]	0·351
	Steps	31/26	9242·09 (3317·87) [8734·17]	-239·05 [-1743·88 to 1265·79]	-0·09 [-0·63 to 0·46]	0·751
	VO_2 Max	46/47	1·81 (0·65) [1·72]	-0·14 [-0·33 to 0·06]	-0·32 [-0·74 to 0·12]	0·153
**Serum**	Calprotectin	54/61	5·68 (4·73) [4·45]	-0·50 [-1·52 to 0·52]	0·19 [-0·2 to 0·57]	0·333
	IL-6	54/61	5·39 (5·25) [3·80]	-1·41 [-3·83 to 1·02]	0·23 [-0·16 to 0·60]	0·253
**Sputum**	24 Hour Weight	27/22	10·97 (11·73) [5·92]	-2·97 [-7·98 to 2·05]	0·37 [-0·26 to 0·98]	0·239
	Total Cells	27/25	13e6 (9e6) [15e6]	3e6 [-3e6 to 9e6]	-0·29 [-0·86 to 0·29]	0·327
	%Eosinophils	23/20	2·54 (7·12) [1·00]	-0·34 [-1·18 to 0·51]	0·26 [-0·39 to 0·90]	0·421
	%Lymphocytes	23/20	0·16 (0·31) [0·00]	-0·04 [-0·12 to 0·06]	0·22 [-0·42 to 0·85]	0·494
	%Macrophages	23/20	3·86 (2·77) [3·25]	-0·59 [-2·89 to 1·72]	-0·17 [-0·81 to 0·48]	0·608
	%Neutrophils	16/28	93·54 (7·26) [95·21]	-2·92 [-6·06 to 0·23]	0·65 [-0·06 to 1·35]	0·068
	Calprotectin	27/25	630·75 (325·32) [556·2]	28·94 [-203·3 to 261·17]	-0·08 [-0·65 to 0·50]	0·803
	IL-8	27/23	28·67 (18·86) [25·33]	-0·84 [-8·06 to 6·39]	0·07 [-0·53 to 0·67]	0·817

Alton *et al* Figure S6

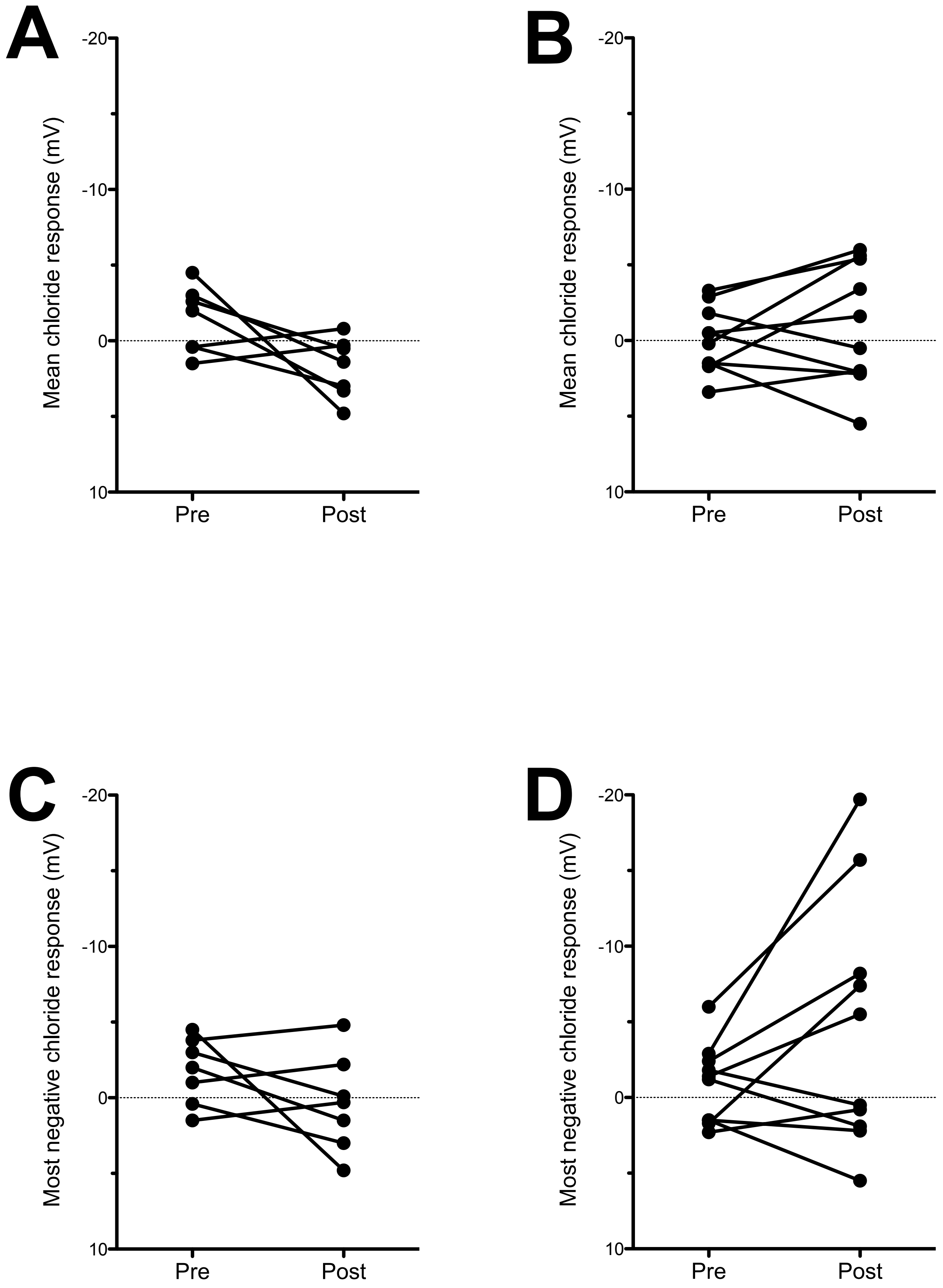
A
B
C
D
Mean chloride response (mV)
Most negative chloride response (mV)
-20
-10
0
10
Pre
Post

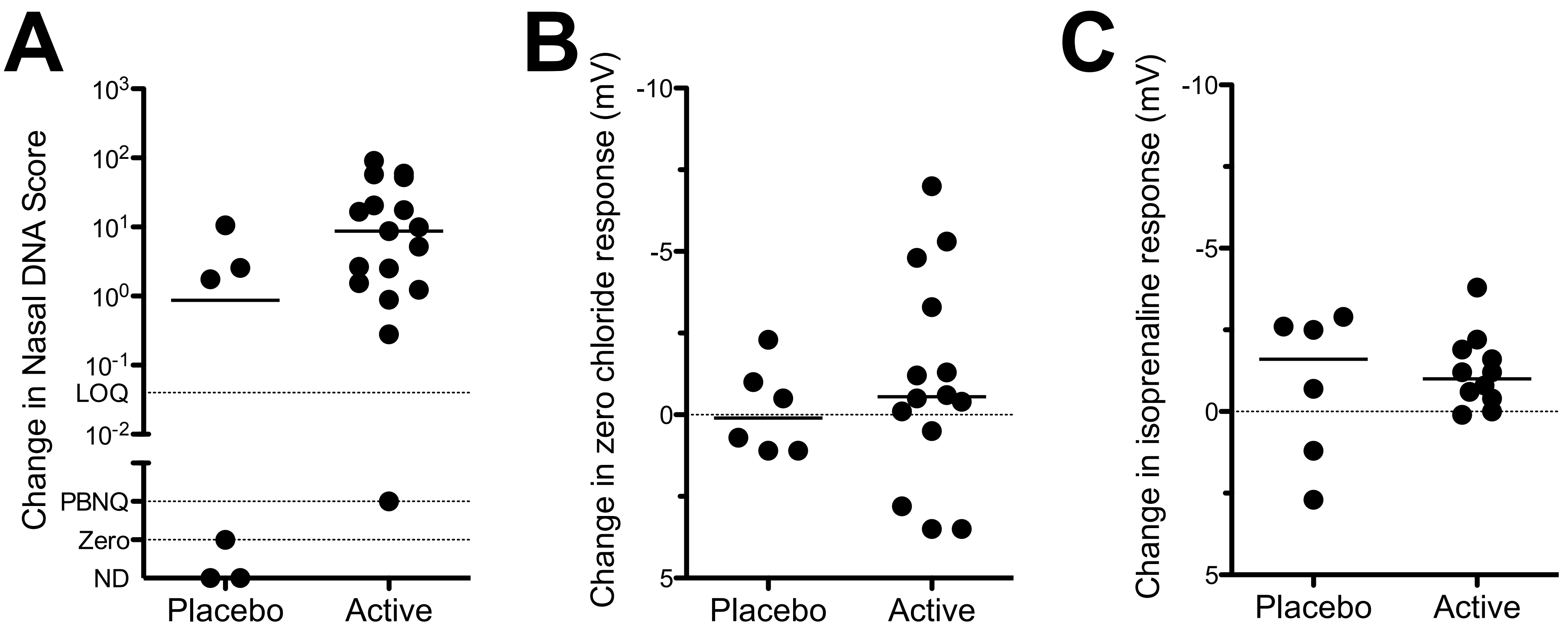

Alton *et al* Figure S8

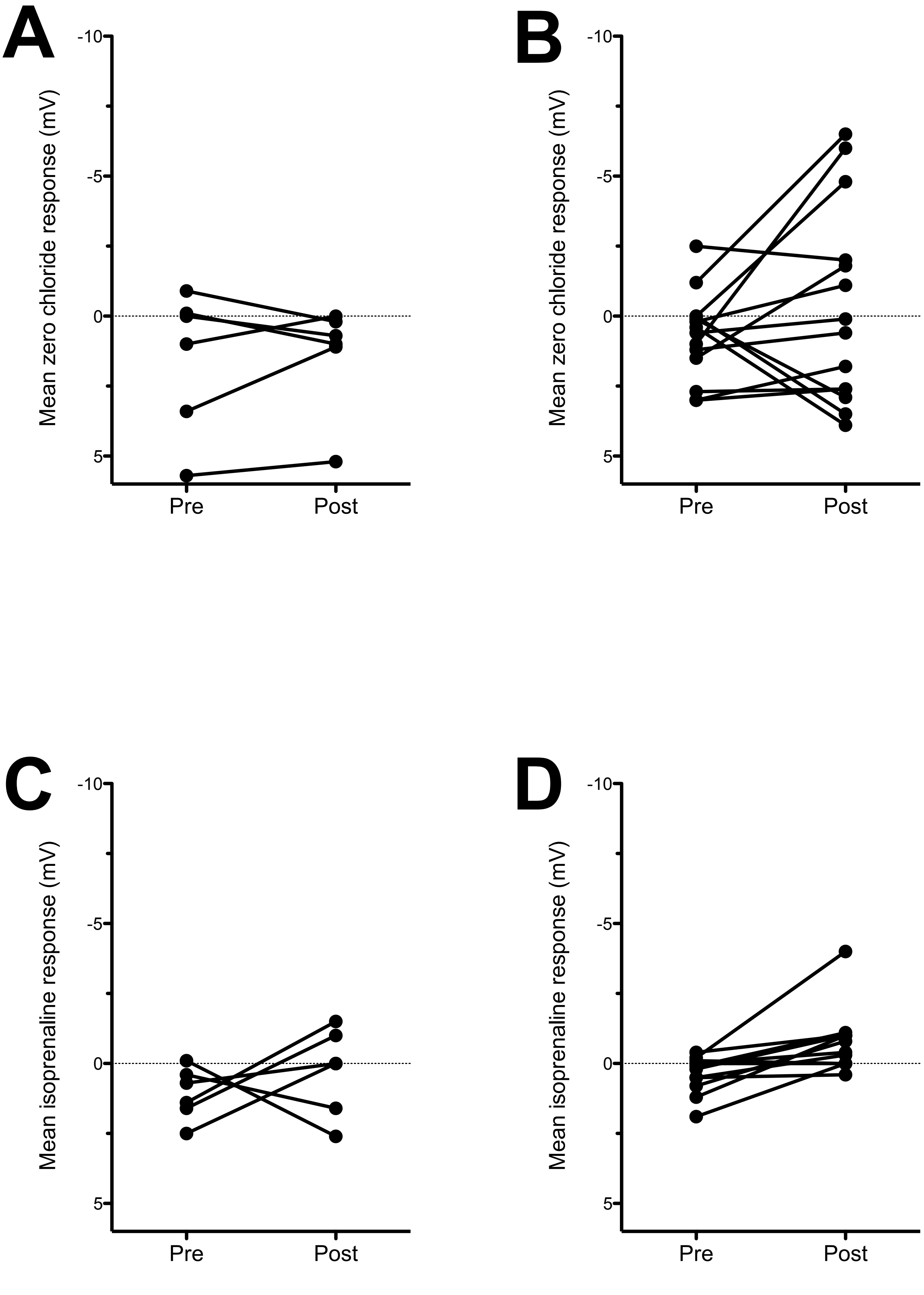
A
B
C
D
Mean zero chloride response (mV)
Mean isoprenaline response (mV)
-10
-5
0
5
Pre
Post

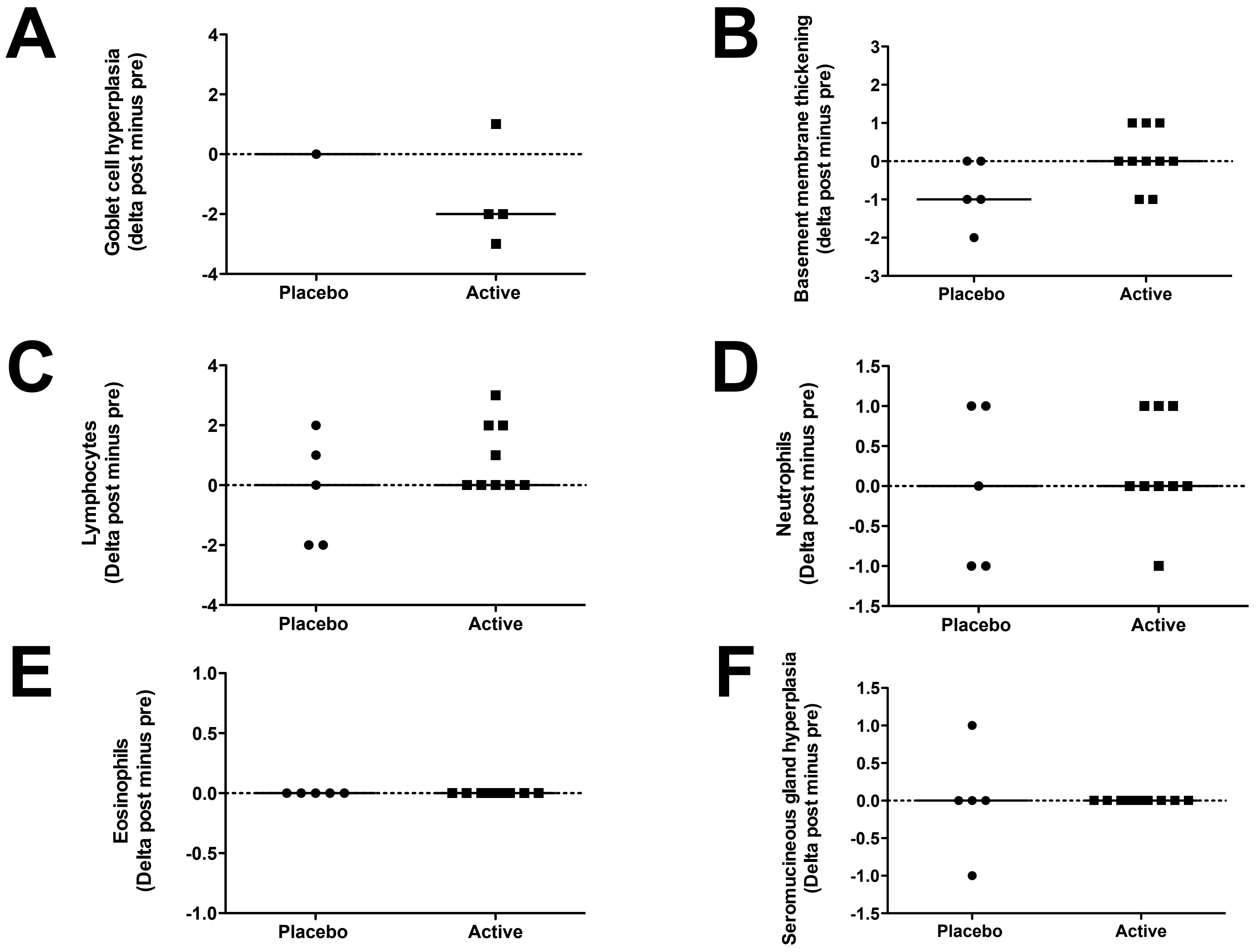

Alton *et al* Figure S10

1	Admission to hospital with acute pancreatitis: 47 year old pancreatic sufficient male with multiple previous episodes of acute, non-alcohol related pancreatitis. Managed conservatively. 4 weeks after last dose.
2	Admission to hospital with severe headache, fever, pulmonary exacerbation and new isolate of MRSA: 28 year old male with flu-like illness within 24 hours of trial bronchoscopy. Hospital admission for IV antibiotics. 5 weeks after last dose.
3	Pneumothorax after removal of indwelling intravenous access device: 23 year old female with semi-elective admission for removal and replacement of malfunctioning Portacath. Small pneumothorax induced during surgery requiring no intervention. 4 days after last dose.
4	Post-surgical infection: 46 year old female undergoing laparascopic Nissen's fundoplication for severe reflux. 10 days post surgery, admitted with pain and fever secondary to surgical site abscess, treated 20 days after last dose.
5	Admission to hospital with headache, vomiting and viral URTI symptoms: 25 year old female. Overnight admission with conservative management. 11 days after previous dose.
6	Admission to hospital with minor vomiting illness: 14 year old male with diabetes. Admitted for assistance with diabetic control. 16 days after previous dose.

	**PP Population**			
	**Placebo (n=54)**		**Active (n=62)**	
**CFTR-Specific T-cells**	**Pre-Dose**	**Follow Up**	**Pre-Dose**	**Follow Up**
**Negative**	33	36	37	49
**Positive**	4	8	1	2
**Not Determined**	17	10	24	11
**T cell conversion**		1*	1**	

**Anti-dsDNA antibody**	**PP Population**			
	**Placebo (n=54)**		**Active (n=62)**	
	**Pre**	**Post**	**Pre**	**Post**
**Negative**	51	51	60	56
**Positive**	0	1	0	0
**Not determined**	3	2	2	6

A

Lipid-Staining Tissue Macrophages	Placebo		Active	
	Pre	Post	Pre	Post
**0**	3	3	9	6
**1-6**	2	2	1	6
**7-14**			2	
**≥15**	1		1	

B

Lipid-Staining Sputum Cells	Mean [95% CI] (n)		Treatment Effect	P Value
	Placebo	Active		
**Macrophages**	-3.27 [-6.60 to -0.05] (24)	-2.13 [-5.45 to 1.20] (24)	1.14 [-3.63 to 5.92]	0.631
**Neutrophils**	-0.23 [-1.62 to 1.16] (19)	0.45 [-0.87 to 1.77] (21)	0.69 [-1.27 to 2.64]	0.480
**Squamous Cells**	-18.24 [-44.26 to 7.78] (5)	-3.49 [-21.23 to 14.25] (9)	14.75 [-20.87 to 50.37]	0.360
